# Current Awareness on Comparative and Functional Genomics

**DOI:** 10.1002/cfg.357

**Published:** 2004-12


**Comparative and Functional Genomics**
*Comp Funct Genom* 2004; **5**: 658–669
Published online in Wiley InterScience (www.interscience.wiley.com). **DOI:** 10.1002cfg.357

### Current awareness on comparative and functional genomics

CURRENT AWARENESS - USE THE LINKS ONLINE

### 1 Reviews & symposia

2004. Special issue - Toxicogenomics. *Mutat Res* **549:** (1-2)

Agaton C, Uhlen M, Hober S. 2004. Genome-based proteomics. *Electrophoresis* **25:** (9) 1280.

Asenjo JA, Andrews BA. 2004. Is there a rational method to purify proteins? From expert systems to proteomics. *J Mol Recognit* **17:** (3) 236.

Austin CP. 2004. The impact of the completed human genome sequence on the development of novel therapeutics for human disease. *Annu Rev Med* **55:** 1.

Baginsky S, Gruissem W. 2004. Chloroplast proteomics: Potentials and challenges. *J Exp Bot* **55:** (400) 1213.

Beck N. 2004. Proteomics in pathology, research and practice: Ethical considerations. *Pathol Res Pract* **200:** (2) 179.

Bell J. 2004. Predicting disease using genomics. *Nature* **429:** (6990) 453.

Benning C, Stitt M. 2004. Physiology and metabolism reacting to the full complexity of metabolic pathways in a postgenomic era. *Curr Opin Plant Biol* **7:** (3) 231.

Bentley DR. 2004. Genomes for medicine. *Nature* **429:** (6990) 440.

Boffelli D, Nobrega MA, Rubin EM. 2004. Comparative genomics at the vertebrate extremes. *Nat Rev Genet* **5:** (6) 456.

Bukowska A, Lendeckel U, Kahne T, Goette A. 2004. Proteomics in myocardial diseases. *Pathol Res Pract* **200:** (2) 135.

Choleris E, Kavaliers M, Pfaff DW. 2004. Functional genomics of social recognition. *J Neuroendocrinol* **16:** (4) 383.

Choudhary J, Grant SGN. 2004. Proteomics in postgenomic neuroscience: The end of the beginning. *Nat Neurosci* **7:** (5) 440.

Clark MS, Clarke A, Cockell CS, Convey P, Detrich HW, Fraser KPP, Johnston IA, Methe BA, Murray AE, Peck LS, Romisch K, Rogers AD. 2004. Antarctic genomics. *Comp Funct Genom* **5:** (3) 230.

Cogburn LA, Wang X, Carre W, Rejto L, Aggrey SE, Duclos MJ, Simon J, Porter TE. 2004. Functional genomics in chickens: Development of integrated-systems microarrays for transcriptional profiling and discovery of regulatory pathways (Conference Review). *Comp Funct Genom* **5:** (3) 253.

Cordonnier-Pratt MM, Liang C, Wang H, Kolychev DS, Sun F, Freeman R, Sullivan R, Pratt LH. 2004. MAGIC Database and interfaces: An integrated package for gene discovery and expression (Conference Review). *Comp Funct Genom* **5:** (3) 268.

Coughlan SJ, Agrawal V, Meyers B. 2004. A comparison of global gene expression measurement technologies in *Arabidopsis thaliana* (Conference Review). *Comp Funct Genom* **5:** (3) 245.

Cowan DA, Arslanoglu A, Burton SG, Baker GC, Cameron RA, Smith JJ, Meyer Q. 2004. Metagenomics, gene discovery and the ideal biocatalyst. *Biochem Soc Trans* **32:** (2) 298.

Cristea IM, Gaskell SJ, Whetton AD. 2004. Proteomics techniques and their application to hematology (Review). *Blood* **103:** (10) 3624.

Cutillas P, Burlingame A, Unwin R. 2004. Proteomic strategies and their application in studies of renal function. *News Physiol Sci* **19:** 114.

De Longueville F, Bertholet V, Remacle J. 2004. DNA microarrays as a tool in toxicogenomics. *Comb Chem High Throughput Scr* **7:** (3) 207.

Edwards D, Batley J. 2004. Plant bioinformatics: From genome to phenome (Review). *Trends Biotechnol* **22:** (5) 232.

Ellis J, Sinclair D, Morrison D. 2004. Microarrays and stage conversion in *Toxoplasma gondii* (Review). *Trends Parasitol* **20:** (6) 288.

Evans WE, Relling MV. 2004. Moving towards individualized medicine with pharmacogenomics. *Nature* **429:** (6990) 464.

Felley-Bosco E, Andre M. 2004. Proteomics and chronic inflammatory bowel diseases. *Pathol Res Pract* **200:** (2) 129.

Galperin MY. 2004. Bacterial signal transduction network in a genomic perspective (Review). *Environ Microbiol* **6:** (6) 552.

Goodacre R, Vaidyanathan S, Dunn WB, Harrigan GG, Kell DB. 2004. Metabolomics by numbers: Acquiring and understanding global metabolite data (Review). *Trends Biotechnol* **22:** (5) 245.

Gurtler V, Mayall BC, Seviour R. 2004. Can whole genome analysis refine the taxonomy of the genus *Rhodococcus*? *FEMS Microbiol Rev* **28:** (3) 377.

Hennebold JD. 2004. Characterization of the ovarian transcriptome through the use of differential analysis of gene expression methodologies (Review). *Hum Reprod Update* **10:** (3) 227.

Hirsch J, Hansen KC, Burlingame AL, Matthay MA. 2004. Proteomics: Current techniques and potential applications to lung disease (Review). *Am J Physiol* **287:** (1) L1.

Hu W, Brindley PJ, McManus DP, Feng Z, Han ZG. 2004. Schistosome transcriptomes: New insights into the parasite and schistosomiasis (Review). *Trends Mol Med* **10:** (5) 217.

Julka S, Regnier F. 2004. Quantification in proteomics through stable isotope coding: A review. *J Proteome Res* **3:** (3) 350.

Kapur G, Mattoo T, Aranda JV. 2004. Pharmacogenomics and renal drug disposition in the newborn. *Semin Perinatol* **28:** (2) 132.

Kennedy GC, Wilson IW. 2004. Plant functional genomics: Opportunities in microarray databases and data mining (Review). *Funct Plant Biol* **31:** (4) 295.

In order to keep subscribers up-to-date with the latest developments in their field, this current awareness service is provided by John Wiley & Sons and contains newly-published material on comparative and functional genomics. Each bibliography is divided into 16 sections. 1 Reviews & symposia; 2 General; 3 Large-scale sequencing and mapping; 4 Evolutionary genomics; 5 Comparative genomics; 6 Pathways, gene families and regulons; 7 Pharmacogenomics; 8 EST, cDNA and other clone resources; 9 Functional genomics; 10 Transcriptomics; 11 Proteomics; 12 Protein structural genomics; 13 Metabolomics; 14 Genomic approaches to development; 15 Technological advances; 16 Bioinformatics. Within each section, articles are listed in alphabetical order with respect to author. If, in the preceding period, no publications are located relevant to any one of these headings, that section will be omitted.

Copyright © 2005 John Wiley & Sons, Ltd.

Kim AS. 2004. Clinical impact of gene expression profiling on oncology diagnosis, prognosis, and treatment. *Comb Chem High Throughput Scr* **7:** (3) 183.

Kley N. 2004. Chemical dimerizers and three-hybrid systems: Scanning the proteome for targets of organic small molecules (Review). *Chem Biol* **11:** (5) 599.

Knapp S, Bohs L, Nee M, Spooner DM. 2004. Solanaceae - A model for linking genomics with biodiversity (Conference Review). *Comp Funct Genom* **5:** (3) 285.

Kosak S, Groudine M. 2004. Form follows function: The genomic organization of cellular differentiation (Review). *Genes Dev* **18:** (12) 1371.

Krenn V, Petersen I, Haupl T, Koepenik A, Blind C, Dietel M, Konthur Z, Skriner K. 2004. Array technology and proteomics in autoimmune diseases. *Pathol Res Pract* **200:** (2) 95.

Lage H. 2004. Proteomics in cancer cell research: An analysis of therapy resistance. *Pathol Res Pract* **200:** (2) 105.

Lescuyer P, Hochstrasser DF, Sanchez JC. 2004. Comprehensive proteome analysis by chromatographic protein prefractionation. *Electrophoresis* **25:** (7-8) 1125.

Lescuyer P, Chevallet M, Rabilloud T. 2004. Concepts and therapeutic perspectives of proteomics (French, English Abstract). *Med Sci (Paris)* **20:** (5) 587.

Lohr M, Faissner R. 2004. Proteomics in pancreatic disease (Review). *Pancreatology* **4:** (2) 67.

Lord PG. 2004. Progress in applying genomics in drug development. *Toxicol Lett* **149:** (1-3) 371.

Love CG, Batley J, Lim G, Robinson AJ, Savage D, Singh D, Spangenberg GC, Edwards D. 2004. New computational tools for *Brassica* genome research (Conference Review). *Comp Funct Genom* **5:** (3) 276.

Matthew L. 2004. RNAi for plant functional genomics (Conference Review). *Comp Funct Genom* **5:** (3) 240.

McGrath S, Fitzgerald GF, Van Sinderen D. 2004. The impact of bacteriophage genomics. *Curr Opin Biotechnol* **15:** (2) 94.

Meyers BC, Galbraith DW, Nelson T, Agrawal V. 2004. Methods for transcriptional profiling in plants. Be fruitful and replicate. *Plant Physiol* **135:** (2) 637.

Mirnics K, Pevsner J. 2004. Progress in the use of microarray technology to study the neurobiology of disease. *Nat Neurosci* **7:** (5) 434.

Nair KS, Jaleel A, Asmann YW, Short KR, Raghavakaimal S. 2004. Proteomic research: Potential opportunities for clinical and physiological investigators (Review). *Am J Physiol* **286:** (6) E863.

Nes IF, Johnsborg A. 2004. Exploration of antimicrobial potential in LAB by genomics. *Curr Opin Biotechnol* **15:** (2) 100.

Neumann E, Gay RE, Gay S, Muller-Ladner U. 2004. Functional genomics of fibroblasts. *Curr Opin Rheumatol* **16:** (3) 238.

Oliveira G, Rodrigues NB, Romanha AJ, Bahia D. 2004. Genome and genomics of schistosomes. *Can J Zool* **82:** (2) 375.

Petty RD, Nicolson MC, Kerr KM, Collie-Duguid E, Murray GI. 2004. Gene expression profiling in non-small cell lung cancer: From molecular mechanisms to clinical application (Review). *Clin Cancer Res* **10:** (10) 3237.

Reymond MA, Steinert R, Kahne T, Sagynaliev E, Allal AS, Lippert H. 2004. Expression and functional proteomics studies in colorectal cancer. *Pathol Res Pract* **200:** (2) 119.

Righetti PG, Campostrini N, Pascali J, Astner MHH. 2004. Quantitative proteomics: A review of different methodologies. *Eur J Mass Spectrom* **10:** (3) 335.

Rocken C, Ebert MPA, Roessner A. 2004. Proteomics in pathology, research and practice. *Pathol Res Pract* **200:** (2) 69.

Rogatcheva MM, Rund LA, Swanson KS, Marron BM, Beever JE, Counter CM, Schook LB. 2004. Creating porcine biomedical models through recombineering (Conference Review). *Comp Funct Genom* **5:** (3) 262.

Rosenblatt KP, Bryant-Greenwood P, Killian JK, Mehta A, Geho D, Espina V, Petricoin EE, Liotta LA. 2004. Serum proteomics in cancer diagnosis and management. *Annu Rev Med* **55:** 97.

Schneider G, Fechner U. 2004. Advances in the prediction of protein targeting signals. *Proteomics* **4:** (6) 1571.

Seibert V, Wiesner A, Buschmann T, Meuer J. 2004. Surface-enhanced laser desorption ionization time-of-flight mass spectrometry (SELDI TOF-MS) and ProteinChip® technology in proteomics research (Review). *Pathol Res Pract* **200:** (2) 83.

Siezen RJ, Van Enckevort FH, Kleerebezem M, Teusink B. 2004. Genome data mining of lactic acid bacteria: The impact of bioinformatics. *Curr Opin Biotechnol* **15:** (2) 105.

Strausberg RL, Simpson AJG, Old LJ, Riggins GJ. 2004. Oncogenomics and the development of new cancer therapies. *Nature* **429:** (6990) 469.

Swigonova Z, Lai J, Ma J, Ramakrishna W, Llaca V, Bennetzen JL, Messing J. 2004. On the tetraploid origin of the maize genome (Conference Review). *Comp Funct Genom* **5:** (3) 281.

Tjalsma H, Antelmann H, Jongbloed JDH, Braun PG, Darmon E, Dorenbos R, Dubois JYF, Westers H, Zanen G, Quax WJ *et al*. 2004. Proteomics of protein secretion by *Bacillus subtilis*: Separating the "secrets" of the secretome. *Microbiol Mol Biol Rev* **68:** (2) 207.

Waldburg N, Kahne T, Reisenauer A, Rocken C, Welte T, Buhling F. 2004. Clinical proteomics in lung diseases. *Pathol Res Pract* **200:** (2) 147.

Walgren JL, Thompson DC. 2004. Application of proteomic technologies in the drug development process. *Toxicol Lett* **149:** (1-3) 377.

Weems D, Miller N, Garcia-Hernandez M, Huala E, Rhee SY. 2004. Design, implementation and maintenance of a model organism database for *Arabidopsis thaliana* (Review). *Comp Funct Genom* **5:** (4) 362.

Weeraratna AT, Nagel JE, De Mello-Coelho V, Taub DD. 2004. Gene expression profiling: From microarrays to medicine. *J Clin Immunol* **24:** (3) 213.

Wen F, Li F, Xia HY, Lu X, Zhang XG, Li YD. 2004. The impact of very short alternative splicing on protein structures and functions in the human genome. *Trends Genet* **20:** (5) 232.

Wen YM. 2004. Structural and functional analysis of full-length hepatitis B virus genomes in patients: Implications in pathogenesis (Review). *J Gastroenterol Hepatol* **19:** (5) 485.

Werner T. 2004. Proteomics and regulomics: The Yin and Yang of functional genomics. *Mass Spectrom Rev* **23:** (1) 25.

Wiemer JC, Prokudin A. 2004. Bioinformatics in proteomics: Application, terminology, and pitfalls. *Pathol Res Pract* **200:** (2) 173.

Wollscheid B, Watts JD, Aebersold R. 2004. Proteomics/genomics and signaling in lymphocytes. *Curr Opin Immunol* **16:** (3) 337.

### 2 General

Rinaldi A. 2004. A new code for life - Expansion of the genetic code and artificial assembly of functional genomes are raising important questions about ethics and safety. *EMBO Rep* **5:** (4) 336.

### 3 Large-scale sequencing and mapping

Deloukas P, Earthrowl ME, Grafham DV, Rubenfield M, French L, Steward CA, Sims SK, Jones MC, Searle S, Scott C *et al*. 2004. The DNA sequence and comparative analysis of human chromosome 10. *Nature* **429:** (6990) 375.

Futterer O, Angelov A, Liesegang H, Gottschalk G, Schleper C, Schepers B, Dock C, Antranikian G, Liebl W. 2004. Genome sequence of *Picrophilus torridus* and its implications for life around pH 0. *Proc Natl Acad Sci U S A* **101:** (24) 9091.

Heidelberg JF, Seshadri R, Haveman SA, Hemme CL, Paulsen IT, Kolonay JF, Eisen JA, Ward N, Methe B, Brinkac LM *et al*. 2004. The genome sequence of the anaerobic, sulfate-reducing bacterium *Desulfovibrio vulgaris* Hildenborough. *Nat Biotechnol* **22:** (5) 554.

Henne A, Bruggemann H, Raasch C, Wiezer A, Hartsch T, Liesegang H, Johann A, Lienard T, Gohl O, Martinez-Arias R *et al*. 2004. The genome sequence of the extreme thermophile *Thermus thermophilus*. *Nat Biotechnol* **22:** (5) 547.

Humphray SJ, Oliver K, Hunt AR, Plumb RW, Loveland JE, Howe KL, Andrews TD, Searle S, Hunt SE, Scott CE *et al*. 2004. DNA sequence and analysis of human chromosome 9. *Nature* **429:** (6990) 369.

Jones T, Federspiel NA, Chibana H, Dungan J, Kalman S, Magee BB, Newport G, Thorstenson YR, Agabian N, Magee PT, Davies RW, Scherer S. 2004. The diploid genome sequence of *Candida albicans*. *Proc Natl Acad Sci U S A* **101:** (19) 7329.

Martinez D, Larrondo LF, Putnam N, Gelpke MDS, Huang K, Chapman J, Helfenbein KG, Ramaiya P, Detter JC, Larimer F *et al*. 2004. Genome sequence of the lignocellulose degrading fungus *Phanerochaete chrysosporium* strain RP78. *Nat Biotechnol* **22:** (6) 695.

Copyright © 2005 John Wiley & Sons, Ltd.

Mita K, Kasahara M, Sasaki S, Nagayasu Y, Yamada T, Kanamori H, Namiki N, Kitagawa M, Yamashita H, Yasukochi Y *et al*. 2004. The genome sequence of silkworm, *Bombyx mori*. *DNA Res* **11:** (1) 27.

Nievergelt CM, Smith DW, Kohlenberg JB, Schork NJ. 2004. Large-scale integration of human genetic and physical maps. *Genome Res* **14:** (6) 1199.

Schmutz J, Wheeler J, Grimwood J, Dickson M, Yang DJ, Caoile C, Bajorek E, Black S, Chan YM, Denys M *et al*. 2004. Quality assessment of the human genome sequency. *Nature* **429:** (6990) 365.

Watanabe H, Fujiyama A, Hattori M, Taylor TD, Toyoda A, Kuroki Y, Noguchi H, BenKahla A, Lehrach H, Sudbrak R *et al*. 2004. DNA sequence and comparative analysis of chimpanzee chromosome 22. *Nature* **429:** (6990) 382.

### 4 Evolutionary genomics

Brugger K, Torarinsson E, Redder P, Chen L, Garrett RA. 2004. Shuffling of *Sulfolobus* genomes by autonomous and non-autonomous mobile elements. *Biochem Soc Trans* **32:** (2) 179.

Castillo-Davis CI, Kondrashov FA, Hartl DL, Kulathinal RJ. 2004. The functional genomic distribution of protein divergence in two animal phyla: Coevolution, genomic conflict, and constraint. *Genome Res* **14:** (5) 802.

Christoffels A, Koh EGL, Chia JM, Brenner S, Aparicio S, Venkatesh B. 2004. Fugu genome analysis provides evidence for a whole-genome duplication early during the evolution of ray-finned fishes. *Mol Biol Evol* **21:** (6) 1146.

Dujon B, Sherman D, Fischer G, Durrens P, Casaregola S, Lafontaine I, De Montigny J, Marck C, Neuveglise C, Talla E *et al*. 2004. Genome evolution in yeasts. *Nature* **430:** (6995) 35.

Gu Y, Coleman-Derr D, Kong X, Anderson O. 2004. Rapid genome evolution revealed by comparative sequence analysis of orthologous regions from four Triticeae genomes. *Plant Physiol* **135:** (1) 459.

Jabbari K, Bernardi G. 2004. Body temperature and evolutionary genomics of vertebrates: A lesson from the genomes of *Takifugu rubripes* and *Tetraodon nigroviridis*. *Gene* **333:** 179.

Jakob SS, Meister A, Blattner FR. 2004. Considerable genome size variation of *Hordeum* species (Poaceae) is linked to phylogeny, life form, ecology, and speciation rates. *Mol Biol Evol* **21:** (5) 860.

Klenk HP, Spitzer M, Ochsenreiter T, Fuellen G. 2004. Phylogenomics of hyperthermophilic Archaea and bacteria. *Biochem Soc Trans* **32:** (2) 175.

Lercher MJ, Chamary JV, Hurst LD. 2004. Genomic regionality in rates of evolution is not explained by clustering of genes of comparable expression profile. *Genome Res* **14:** (6) 1002.

Nguyen D, Brassard P, Menzies D, Thibert L, Warren R, Mostowy S, Behr M. 2004. Genomic characterization of an endemic *Mycobacterium tuberculosis* strain: Evolutionary and epidemiologic implications. *J Clin Microbiol* **42:** (6) 2573.

Simillion C, Vandepoele K, Saeys Y, De Peer YV. 2004. Building genomic profiles for uncovering segmental homology in the twilight zone. *Genome Res* **14:** (6) 1095.

Sorimachi K, Okayasu T. 2004. Classification of eubacteria based on their complete genome: Where does Mycoplasmataceae belong? *Proc R Soc Lond Ser B Biol Sci* **271:** (Suppl 4) S127.

### 5 Comparative genomics

Bai XD, Zhang JH, Holford IR, Hogenhout SA. 2004. Comparative genomics identifies genes shared by distantly related insect-transmitted plant pathogenic mollicutes. *FEMS Microbiol Lett* **235:** (2) 249.

Boivin K, Acarkan A, Mbulu RS, Clarenz O, Schmidt R. 2004. The *Arabidopsis* genome sequence as a tool for genome analysis in Brassicaceae. A comparison of the *Arabidopsis* and *Capsella rubella* genomes. *Plant Physiol* **135:** (2) 735.

Bourdeau V, Deschenes J, Metivier R, Nagai Y, Nguyen D, Bretschneider N, Gannon F, White JH, Mader S. 2004. Genome-wide identification of high-affinity estrogen response elements in human and mouse. *Mol Endocrinol* **18:** (6) 1411.

Comfort D, Clubb RT. 2004. Comparative genome analysis identifies distinct sorting pathways in Gram-positive bacteria. *Infect Immun* **72:** (5) 2710.

Hagiwara D, Yamashino T, Mizuno T. 2004. Genome-wide comparison of the His-to-Asp phosphorelay signaling components of three symbiotic genera of rhizobia. *DNA Res* **11:** (1) 57.

Jabbari K, Bernardi G. 2004. Comparative genomics of *Anopheles gambiae* and *Drosophila melanogaster*. *Gene* **333:** 183.

Nelson DR, Schuler MA, Paquette SM, Werck-Reichhart D, Bak S. 2004. Comparative genomics of rice and *Arabidopsis*. Analysis of 727 cytochrome P450 genes and pseudogenes from a monocot and a dicot. *Plant Physiol* **135:** (2) 756.

Tang JB, Xia HA, Cao ML, Zhang XQ, Zeng WY, Hu SN, Tong W, Wang J, Wang J, Yu J *et al*. 2004. A comparison of rice chloroplast genomes. *Plant Physiol* **135:** (1) 412.

Brugger K, Torarinsson E, Redder P, Chen L, Garrett RA. 2004. Shuffling of *Sulfolobus* genomes by autonomous and non-autonomous mobile elements. *Biochem Soc Trans* **32:** (2) 179.

Simillion C, Vandepoele K, Saeys Y, De Peer YV. 2004. Building genomic profiles for uncovering segmental homology in the twilight zone. *Genome Res* **14:** (6) 1095.

Zhang CT, Zhang R. 2004. A nucleotide composition constraint of genome sequences. *Comput Biol Chem* **28:** (2) 149.

Yap MN, Barak JD, Charkowski AO. 2004. Genomic diversity of *Erwinia carotovora* subsp *carotovora* and its correlation with virulence. *Appl Environ Microbiol* **70:** (5) 3013.

Zagrobelny M, Jeffares DC, Arctander P. 2004. Differences in non-LTR retrotransposons within *C. elegans* and *C. briggsae* genomes. *Gene* **330:** 61.

Zhang CT, Zhang R. 2004. A nucleotide composition constraint of genome sequences. *Comput Biol Chem* **28:** (2) 149.

### 6 Pathways, gene families and regulons

Lu CD, Yang Z, Li W. 2004. Transcriptome analysis of the ArgR regulon in *Pseudomonas aeruginosa*. *J Bacteriol* **186:** (12) 3855.

Sam-Yellowe TY, Florens L, Johnson JR, Wang TM, Drazba JA, Le Roch KG, Zhou YY, Batalov S, Carucci DJ, Winzeler EA *et al*. 2004. A *Plasmodium* gene family encoding Maurer's cleft membrane proteins: Structural properties and expression profiling. *Genome Res* **14:** (6) 1052.

Vitreschak AG, Lyubetskaya EV, Shirshin MA, Gelfand MS, Lyubetsky VA. 2004. Attenuation regulation of amino acid biosynthetic operons in proteobacteria: Comparative genomics analysis. *FEMS Microbiol Lett* **234:** (2) 357.

### 7 Pharmacogenomics

Aigner T, Finger F, Zien A, Bartnik E. 2004. cDNA-microarray technology in cartilage research - Functional genomics of osteoarthritis (German, English Abstract). *Z Orthop Ihre Grenzgeb* **142:** (2) 241.

Baek HY, Lim JW, Kim H, Kim JM, Kim JS, Jung HC, Kim KH. 2004. Oxidative-stress-related proteome changes in *Helicobacter pylori*-infected human gastric mucosa. *Biochem J* **379:** (2) 291.

Barella L, Muller PY, Schlachter M, Hunziker W, Stocklin E, Spitzer V, Meier N, De Pascual-Teresa S, Minihane AM, Rimbach G. 2004. Identification of hepatic molecular mechanisms of action of $\alpha$-tocopherol using global gene expression profile analysis in rats. *Biochim Biophys Acta* **1689:** (1) 66.

Barnidge DR, Goodmanson MK, Klee GG, Muddiman DC. 2004. Absolute quantification of the model biomarker prostate-specific antigen in serum by LC-MS/MS using protein cleavage and isotope dilution mass spectrometry. *J Proteome Res* **3:** (3) 644.

Belhocine TZ, Tait JF, Vanderheyden JL, Li C, Blankenberg FG. 2004. Nuclear medicine in the era of genomics and proteomics: Lessons from annexin V. *J Proteome Res* **3:** (3) 345.

Bernstein H, Payne CM, Kunke K, Crowley-Weber CL, Waltmire CN, Dvorakova K, Holubec H, Bernstein C, Vaillancourt RR, Raynes DA *et al*. 2004. A proteomic study of resistance to deoxycholate-induced apoptosis. *Carcinogenesis* **25:** (5) 681.

Bowler RP, Duda B, Chan ED, Enghild JJ, Ware LB, Matthay MA, Duncan MW. 2004. Proteomic analysis of pulmonary edema fluid and plasma in patients with acute lung injury. *Am J Physiol* **286:** (6) L1095.

Copyright © 2005 John Wiley & Sons, Ltd.

Brill LM, Salomon AR, Ficarro SB, Mukherji M, Stettler-Gill M, Peters EC. 2004. Robust phosphoproteomic profiling of tyrosine phosphorylation sites from human T cells using immobilized metal affinity chromatography and tandem mass spectrometry. *Anal Chem* **76:** (10) 2763.
Brown KJ, Fenselau C. 2004. Investigation of doxorubicin resistance in MCF-7 breast cancer cells using shot-gun comparative proteomics with proteolytic $^{18}O$ labeling. *J Proteome Res* **3:** (3) 455.
Cadieux PA, Beiko DT, Watterson JD, Burton JP, Howard JC, Knudsen BE, Gan BS, McCormick JK, Chambers AF, Denstedt JD, Reid G. 2004. Surface-enhanced laser desorption/ionization-time of flight-mass spectrometry (SELDI-TOF-MS): A new proteomic urinary test for patients with urolithiasis. *J Clin Lab Anal* **18:** (3) 170.
Carlson CS, Eberle MA, Kruglyak L, Nickerson DA. 2004. Mapping complex disease loci in whole-genome association studies. *Nature* **429:** (6990) 446.
Casado B, Pannell LK, Viglio S, Iadarola P, Baraniuk JN. 2004. Analysis of the sinusitis nasal lavage fluid proteome using capillary liquid chromatography interfaced to electrospray ionization-quadrupole time of flight-tandem mass spectrometry. *Electrophoresis* **25:** (9) 1386.
Chandra BR, Gowthaman R, Akhouri RR, Gupta D, Sharma A. 2004. Distribution of proline-rich (PxxP) motifs in distinct proteomes: Functional and therapeutic implications for malaria and tuberculosis. *Protein Eng* **17:** (2) 175.
Chiaretti S, Li XC, Gentleman R, Vitale A, Vignetti M, Mandelli F, Ritz J, Foa R. 2004. Gene expression profile of adult T-cell acute lymphocytic leukemia identifies distinct subsets of patients with different response to therapy and survival. *Blood* **103:** (7) 2771.
Coldham NG, Woodward MJ. 2004. Characterization of the *Salmonella typhimurium* proteome by semi-automated two dimensional HPLC-mass spectrometry: Detection of proteins implicated in multiple antibiotic resistance. *J Proteome Res* **3:** (3) 595.
Dhondt G, Vanrobaeys F, Devreese B, Van Beeumen J, Van Coster R. 2004. Proteomic identification of the myelin proteins. *Acta Neurol Belg* **104:** (1) 47.
Drynda S, Ringel B, Kekow M, Kuhne C, Drynda A, Glocker MO, Thiesen HJ, Kekow J. 2004. Proteome analysis reveals disease-associated marker proteins to differentiate RA patients from other inflammatory joint diseases with the potential to monitor anti-TNFα therapy. *Pathol Res Pract* **200:** (2) 165.
Duan X, Yarmush DM, Jayaraman A, Yarmush ML. 2004. Dispensable role for interferon-γ in the burn-induced acute phase response: A proteomic analysis. *Proteomics* **4:** (6) 1830.
Enk CD, Shahar I, Amariglio N, Rechavi G, Kaminski N, Hochberg M. 2004. Gene expression profiling of *in vivo* UVB-irradiated human epidermis. *Photodermatol Photoimmunol Photomed* **20:** (3) 129.
Eustace BK, Sakurai T, Stewart JK, Yimlamai D, Unger C, Zehetmeier C, Lain B, Torella C, Henning SW, Beste G *et al*. 2004. Functional proteomic screens reveal an essential extracellular role for hsp90α in cancer cell invasiveness. *Nat Cell Biol* **6:** (6) 507.
Ezendam J, Staedtler F, Pennings J, Vandebriel RJ, Pieters R, Harleman JH, Vos JG. 2004. Toxicogenomics of subchronic hexachlorobenzene exposure in brown Norway rats. *Environ Health Perspect* **112:** (7) 782.
Feezor RJ, Baker HV, Xiao WH, Lee WA, Huber TS, Mindrinos M, Kim RA, Ruiz-Taylor L, Moldawer LL, Davis RW *et al*. 2004. Genomic and proteomic determinants of outcome in patients undergoing thoracoabdominal aortic aneurysm repair. *J Immunol* **172:** (11) 7103.
Florens L, Liu X, Wang YF, Yang SG, Schwartz O, Peglar M, Carucci DJ, Yates JR, Wu YM. 2004. Proteomics approach reveals novel proteins on the surface of malaria-infected erythrocytes. *Mol Biochem Parasitol* **135:** (1) 1.
Fransson J, Ek S, Ellmark P, Soderlind E, Borrebaeck CAK, Furebring C. 2004. Profiling of internalizing tumor-associated antigens on breast and pancreatic cancer cells by reversed genomics. *Cancer Lett* **208:** (2) 235.
Fries RS, Mahboubi P, Mahapatra NR, Mahata SK, Schork NJ, Schmid-Schoenbein GW, O'Connor DT. 2004. Neuroendocrine transcriptome in genetic hypertension - Multiple changes in diverse adrenal physiological systems. *Hypertension* **43:** (6) 1301.
Fujimoto T, Nishikawa A, Iwasaki M, Akutagawa N, Teramoto M, Kudo R. 2004. Gene expression profiling in two morphologically different uterine cervical carcinoma cell lines derived from a single donor using a human cancer cDNA array. *Gynecol Oncol* **93:** (2) 446.
Gangnus R, Langer S, Breit E, Pantel K, Speicher MR. 2004. Genomic profiling of viable and proliferative micrometastatic cells from early-stage breast cancer patients. *Clin Cancer Res* **10:** (10) 3457.
Gaucher SP, Taylor SW, Fahy E, Zhang B, Warnock DE, Ghosh SS, Gibson BW. 2004. Expanded coverage of the human heart mitochondrial proteome using multidimensional liquid chromatography coupled with tandem mass spectrometry. *J Proteome Res* **3:** (3) 495.
Gehrmann ML, Fenselau C, Hathout Y. 2004. Highly altered protein expression profile in the adriamycin resistant MCF-7 cell line. *J Proteome Res* **3:** (3) 403.
Grant GM, Fortney A, Gorreta F, Estep M, Del Giacco L, Van Meter A, Christensen A, Appalla L, Naouar C, Jamison C *et al*. 2004. Microarrays in cancer research. *Anticancer Res* **24:** (2A) 441.
Hammack BN, Fung KYC, Hunsucker SW, Duncan MW, Burgoon MP, Owens GP, Gilden DH. 2004. Proteomic analysis of multiple sclerosis cerebrospinal fluid. *Mult Scler* **10:** (3) 245.
Hardwidge PR, Rodriguez-Escudero I, Goode D, Donohoe S, Eng J, Goodlett DR, Aebersold R, Finlay BB. 2004. Proteomic analysis of the intestinal epithelial cell response to enteropathogenic *Escherichia coli*. *J Biol Chem* **279:** (19) 20127.
Hegmans JPJJ, Bard MPL, Hemmes A, Luider TM, Kleijmeer MJ, Prins JB, Zitvogel L, Burgers SA, Hoogsteden HC, Lambrecht BN. 2004. Proteomic analysis of exosomes secreted by human mesothelioma cells. *Am J Pathol* **164:** (5) 1807.
Heijne WHM, Slitt AL, Van Bladeren PJ, Groten JP, Klaassen CD, Stierum RH, Van Ommen B. 2004. Bromobenzene-induced hepatotoxicity at the transcriptome level. *Toxicol Sci* **79:** (2) 411.
Hishikawa K, Miura S, Marumo T, Yoshioka H, Mori Y, Takato T, Fujita T. 2004. Gene expression profile of human mesenchymal stem cells during osteogenesis in three-dimensional thermoreversible gelation polymer. *Biochem Biophys Res Commun* **317:** (4) 1103.
Hoang CD, D'Cunha J, Tawfic SH, Gruessner AC, Kratzke RA, Maddaus MA. 2004. Expression profiling of non-small cell lung carcinoma identifies metastatic genotypes based on lymph node tumor burden. *J Thorac Cardiovasc Surg* **127:** (5) 1332.
Hong X, Li YW, Hussain M, Sarkar FH. 2004. Gene expression profiling reveals novel targets of estramustine phosphate in prostate cancer cells. *Cancer Lett* **209:** (2) 187.
Huang YY, Franklin J, Gifford K, Roberts BL, Nicolette CA. 2004. A high-throughput proteo-genomics method to identify antibody targets associated with malignant disease. *Clin Immunol* **11:** (2) 202.
Huguet S, Labas V, Duclos-Vallee JC, Bruneel A, Vinh J, Samuel D, Johanet C, Ballot E. 2004. Heterogeneous nuclear ribonucleoprotein A2/B1 identified as an autoantigen in autoimmune hepatitis by proteome analysis. *Proteomics* **4:** (5) 1341.
Ichikawa Y, Hirokawa M, Aiba N, Fujishima N, Komatsuda A, Saitoh H, Kume M, Miura I, Sawada K. 2004. Monitoring the expression profiles of doxorubicin-resistant K562 human leukemia cells by serial analysis of gene expression. *Int J Hematol* **79:** (3) 276.
Imamura T, Kanai F, Kawakami T, Amarsanaa J, Ijichi H, Hoshida Y, Tanaka Y, Ikenoue T, Tateishi K, Kawabe T *et al*. 2004. Proteomic analysis of the TGF-β signaling pathway in pancreatic carcinoma cells using stable RNA interference to silence Smad4 expression. *Biochem Biophys Res Commun* **318:** (1) 289.
Izzotti A, Bagnasco M, Cartiglia C, Longobardi M, De Flora S. 2004. Proteomic analysis as related to transcriptome data in the lung of chromium(VI)-treated rats. *Int J Oncol* **24:** (6) 1513.
Jain SJ, Watson MA, DeBenedetti MK, Hiraki Y, Moley JF, Milbrandt J. 2004. Expression profiles provide insights into early malignant potential and skeletal abnormalities in multiple endocrine neoplasia type 2B syndrome tumors. *Cancer Res* **64:** (11) 3907.
Jalili PR, Dass C. 2004. Proteome analysis in the bovine adrenal medulla using liquid chromatography with tandem mass spectrometry. *Rapid Commun Mass Spectrom* **18:** (17) 1877.
Jarvis JN, Dozmorov I, Jiang KY, Chen YM, Frank MB, Cadwell C, Turner S, Centola M. 2004. Gene expression arrays reveal a rapid return to normal homeostasis in immunologically-challenged trophoblast-like JAR cells. *J Reprod Immunol* **61:** (2) 99.
Johnson MD, Yu LR, Conrads TP, Kinoshita Y, Uo T, Matthews JD, Lee SW, Smith RD, Veenstra TD, Morrison RS. 2004. Proteome analysis of DNA damage-induced neuronal death using high throughput mass spectrometry. *J Biol Chem* **279:** (25) 26685.

Copyright © 2005 John Wiley & Sons, Ltd. 

Kaab S, Barth AS, Margerie D, Dugas M, Gebauer M, Zwermann L, Merk S, Pfeufer A, Steinmeyer K, Bleich M *et al*. 2004. Global gene expression in human myocardium-oligonucleotide microarray analysis of regional diversity and transcriptional regulation in heart failure. *J Mol Med* **82:** (5) 308.

Kaminski N, Krupsky M. 2004. Gene expression patterns, prognostic and diagnostic markers, and lung cancer biology. *Chest* **125:** (Suppl 5) 111S.

Kaur R, Castano I, Cormack BP. 2004. Functional genomic analysis of fluconazole susceptibility in the pathogenic yeast *Candida glabrata*: Roles of calcium signaling and mitochondria. *Antimicrob Agents Chemother* **48:** (5) 1600.

Kellner U, Steinert R, Seibert V, Heim S, Kellner A, Schulz HU, Roessner A, Kruger S, Reymond M. 2004. Epithelial cell preparation for proteomic and transcriptomic analysis in human pancreatic tissue. *Pathol Res Pract* **200:** (2) 155.

Keyvani K, Sachser N, Witte OW, Paulus W. 2004. Gene expression profiling in the intact and injured brain following environmental enrichment. *J Neuropathol Exp Neurol* **63:** (6) 598.

Kiernan UA, Nedelkov D, Tubbs KA, Niederkofler EE, Nelson RW. 2004. Proteomic characterization of novel serum amyloid P component variants from human plasma and urine. *Proteomics* **4:** (6) 1825.

Kim NS, Hahn Y, Oh JH, Lee JY, Oh KJ, Kim JM, Park HS, Kim S, Song KS, Rho SM *et al*. 2004. Gene cataloging and expression profiling in human gastric cancer cells by expressed sequence tags. *Genomics* **83:** (6) 1024.

Kuo WP, Whipple ME, Epstein JB, Jenssen TK, Santos GS, Ohno-Machado L, Sonis ST. 2004. Deciphering gene expression profiles generated from DNA microarrays and their applications in oral medicine. *Oral Surg Oral Med Oral Pathol* **97:** (5) 584.

Kurokawa Y, Matoba R, Nakamori S, Takemasa I, Nagano H, Dono K, Umeshita K, Sakon M, Monden M, Kato K. 2004. PCR-array gene expression profiling of hepatocellular carcinoma. *J Exp Clin Cancer Res* **23:** (1) 135.

Lindahl M, Irander K, Tagesson C, Stahlbom B. 2004. Nasal lavage fluid and proteomics as means to identify the effects of the irritating epoxy chemical dimethylbenzylamine. *Biomarkers* **9:** (1) 56.

Locht C, Antoine R, Raze D, Mielcarek N, Hot D, Lemoine Y, Mascart F. 2004. *Bordetella pertussis*: From functional genomics to intranasal vaccination. *Int J Med Microbiol* **293:** (7-8) 583.

MacAulay C, Lonergan K, Chi B, Zuyderduyn S, Shein J, Tsao M, LeRiche J, Jones S, Marra M, Lam S *et al*. 2004. Serial analysis of gene expression profiles of developmental stages in non-small cell lung carcinoma. *Chest* **125:** (Suppl 5) 97S.

Malmstrom J, Larsen K, Malmstrom L, Tufvesson E, Parker K, Marchese J, Williamson B, Hattan S, Patterson D, Martin S *et al*. 2004. Proteome annotations and identifications of the human pulmonary fibroblast. *J Proteome Res* **3:** (3) 525.

McElwaine S, Mulligan C, Groet J, Spinelli M, Rinaldi A, Denyer G, Mensah A, Cavani S, Baldo C, Dagna-Bricarelli *et al*. 2004. Microarray transcript profiling distinguishes the transient from the acute type of megakaryoblastic leukaemia (M7) in Down's syndrome, revealing PRAME as a specific discriminating marker. *Br J Haematol* **125:** (6) 729.

Melle C, Ernst G, Schimmel B, Bleul A, Koscielny S, Wiesner A, Bogumil R, Moller U, Osterloh D, Halbhuber KJ *et al*. 2004. A technical triade for proteomic identification and characterization of cancer biomarkers. *Cancer Res* **64:** (12) 4099.

Meneses-Lorente G, Guest PC, Lawrence J, Muniappa N, Knowles MR, Skynner HA, Salim K, Cristea I, Mortishire-Smith R, Gaskell SJ *et al*. 2004. A proteomic investigation of drug-induced steatosis in rat liver. *Chem Res Toxicol* **17:** (5) 605.

Mobley JA, Lam YW, Lau KM, Pais VM, L'Esperance JO, Steadman B, Fuster LMB, Blute RD, Taplin ME, Ho SM. 2004. Monitoring the serological proteome: The latest modality in prostate cancer detection. *J Urol* **172:** (1) 331.

Modlich O, Prisack HB, Pitschke G, Ramp U, Ackermann R, Bojar H, Vogeli TA, Grimm MO. 2004. Identifying superficial, muscle-invasive, and metastasizing transitional cell carcinoma of the bladder: Use of cDNA array analysis of gene expression profiles. *Clin Cancer Res* **10:** (10) 3410.

Munson R, Harrison A, Gillaspy A, Ray W, Carson M, Armbruster D, Gipson J, Gipson M, Johnson L, Lewis L *et al*. 2004. Partial analysis of the genomes of two nontypeable *Haemophilus influenzae* otitis media isolates. *Infect Immun* **72:** (5) 3002.

Nagano Y, Nagahori K, Yoshiro F, Hamaguchi Y, Ishikawa T, Ichikawa Y, Togo S, Okazaki Y, Hayashizaki Y, Shimada H. 2004. Gene expression profile analysis of regenerating liver after portal vein ligation in rats by cDNA microarray system. *Liver Int* **24:** (3) 253.

Nakazawa T, Nakajima A, Seki N, Okawa A, Kato M, Moriya H, Amizuka N, Einhorn TA, Yamazaki M. 2004. Gene expression of periostin in the early stage of fracture healing detected by cDNA microarray analysis. *J Orthop Res* **22:** (3) 520.

Oh P, Li Y, Yu JY, Durr E, Krasinska KM, Carver LA, Testa JE, Schnitzer JE. 2004. Subtractive proteomic mapping of the endothelial surface in lung and solid tumours for tissue-specific therapy. *Nature* **429:** (6992) 629.

Parmigiani G, Garrett E, Anbazhagan B, Gabrielson E. 2004. Molecular classification of lung cancer - A cross-platform comparison of gene expression data sets. *Chest* **125:** (Suppl 5) 103S.

Pawitan Y, Bjohle J, Wedren S, Humphreys K, Skoog L, Huang F, Amler L, Shaw P, Hall P, Bergh J. 2004. Gene expression profiling for prognosis using Cox regression. *Stat Med* **23:** (11) 1767.

Pellagatti A, Esoof N, Watkins F, Langford CF, Vetrie D, Campbell LJ, Fidler C, Cavenagh JD, Eagleton H, Gordon P *et al*. 2004. Gene expression profiling in the myelodysplastic syndromes using cDNA microarray technology. *Br J Haematol* **125:** (5) 576.

Prall F, Ostwald C, Nizze H, Barten M. 2004. Expression profiling of colorectal carcinomas using tissue microarrays: Cell cycle regulatory proteins p21, p27, and p53 as immunohistochemical prognostic markers in univariate and multivariate analysis. *Appl Immunohistochem* **12:** (2) 111.

Prucha M, Ruryk A, Boriss H, Moller E, Zazula R, Herold I, Claus RA, Reinhart KA, Deigner P, Russwurm S. 2004. Expression profiling: Toward an application in sepsis diagnostics. *Shock* **22:** (1) 29.

Quade BJ, Wang TY, Sornberger K, Dal Cin P, Mutter GL, Morton CC. 2004. Molecular pathogenesis of uterine smooth muscle tumors from transcriptional profiling. *Gene Chromosomes Cancer* **40:** (2) 97.

Rash JE, Pereda A, Kamasawa N, Furman CS, Yasumura T, Davidson KGV, Dudek FE, Olson C, Li X, Nagy JI. 2004. High-resolution proteomic mapping in the vertebrate central nervous system: Close proximity of connexin35 to NMDA glutamate receptor clusters and co-localization of connexin36 with immunoreactivity for zonula occludens protein-1 (ZO-1). *J Neurocytol* **33:** (1) 131.

Roblick UJ, Hirschberg D, Habermann JK, Palmberg C, Becker S, Kruger S, Gustafsson M, Bruch HP, Franzen B, Ried T *et al*. 2004. Sequential proteome alterations during genesis and progression of colon cancer. *Cell Mol Life Sci* **61:** (10) 1246.

Rutherford RM, Staedtler F, Kehren J, Chibout SD, Joos L, Tamm M, Gilmartin JJ, Brutsche MH. 2004. Functional genomics and prognosis in sarcoidosis - The critical role of antigen presentation. *Sarcoidosis Vasc Diffuse Lung Dis* **21:** (1) 10.

Scheurer SB, Rybak JN, Rosli C, Neri D, Elia G. 2004. Modulation of gene expression by hypoxia in human umbilical cord vein endothelial cells: A transcriptomic and proteomic study. *Proteomics* **4:** (6) 1737.

Schrattenholz A, Schroer K, Chatterjee SS, Koch E. 2004. Proteome analysis reveals a distinct molecular profile of cellular stress following incubation of DDT1-MF2 smooth muscle cells in the presence of a high concentration of hyperforin. *Planta Med* **70:** (4) 342.

Sommer S, Hunzinger C, Schillo S, Klemm M, Biefang-Arndt K, Schwall G, Putter S, Hoelzer K, Schroer K, Stegmann W *et al*. 2004. Molecular analysis of homocysteic acid-induced neuronal stress. *J Proteome Res* **3:** (3) 572.

Spruessel A, Steimann G, Jung M, Lee SA, Carr T, Fentz AK, Spangenberg J, Zornig C, Juhl HH, David KA. 2004. Tissue ischemia time affects gene and protein expression patterns within minutes following surgical tumor excision. *Biotechniques* **36:** (6) 1030.

Takita J, Ishii M, Tsutsumi S, Tanaka Y, Kato K, Toyoda Y, Hanada R, Yamamoto K, Hayashi Y, Aburatani H. 2004. Gene expression profiling and identification of novel prognostic marker genes in neuroblastoma. *Gene Chromosomes Cancer* **40:** (2) 120.

Terasaka S, Aita Y, Inoue A, Hayashi S, Nishigaki M, Aoyagi K, Sasaki H, Wada-Kiyama Y, Sakuma Y, Akaba S *et al*. 2004. Using a customized DNA microarray for expression profiling of the estrogen-responsive genes to evaluate estrogen activity among natural estrogens and industrial chemicals. *Environ Health Perspect* **112:** (7) 773.

Copyright © 2005 John Wiley & Sons, Ltd. 

Thieblemont C, Nasser V, Felman P, Leroy K, Gazzo S, Callet-Bauchu E, Loriod B, Granjeaud S, Gaulard P, Haioun C *et al*. 2004. Small lymphocytic lymphoma, marginal zone B-cell lymphoma, and mantle cell lymphoma exhibit distinct gene-expression profiles allowing molecular diagnosis. *Blood* **103:** (7) 2727.
Tu XL, Huang A, Bae D, Slaughter N, Whitelegge J, Crother T, Bickel PE, Nel A. 2004. Proteome analysis of lipid rafts in Jurkat cells characterizes a raft subset that is involved in NF-κ B activation. *J Proteome Res* **3:** (3) 445.
Venkatraman A, Landar A, Davis AJ, Chamlee L, Sanderson T, Kim H, Page G, Pompilius M, Ballinger S, Darley-Usmar V *et al*. 2004. Modification of the mitochondrial proteome in response to the stress of ethanol-dependent hepatotoxicity. *J Biol Chem* **279:** (21) 22092.
Volmer MW, Radacz Y, Hahn SA, Klein-Scory S, Stuhler K, Zapatka M, Schmiegel W, Meyer HE, Schwarte-Waldhoff I. 2004. Tumor suppressor Smad4 mediates downregulation of the anti-adhesive invasion-promoting matricellular protein SPARC: Landscaping activity of Smad4 as revealed by a "secretome" analysis. *Proteomics* **4:** (5) 1324.
Walter KL, Borczuk AC, Wang LGQ, Assaad AA, Austin JHM, Pearson GD, Shiau MC, Powell CA. 2004. Class prediction of lung nodule gene expression profiles. *Chest* **125:** (Suppl 5) 104S.
Wang DJ, Jensen R, Gendeh G, Williams K, Pallavicini MG. 2004. Proteome and transcriptome analysis of retinoic acid-induced differentiation of human acute promyelocytic leukemia cells, NB4. *J Proteome Res* **3:** (3) 627.
Wang H, Zhou ZM, Xu M, Li JM, Xiao JH, Xu ZY, Sha JH. 2004. A spermatogenesis-related gene expression profile in human spermatozoa and its potential clinical applications. *J Mol Med* **82:** (5) 317.
Warner GC, Reis PP, Jurisica I, Sultan M, Arora S, MacMillan C, Makitie AA, Grenman R, Reid N, Sukhai M *et al*. 2004. Molecular classification of oral cancer by cDNA microarrays identifies overexpressed genes correlated with nodal metastasis. *Int J Cancer* **110:** (6) 857.
Weissinger EM, Wittke S, Kaiser T, Haller H, Bartel S, Krebs R, Golovko I, Rupprecht HD, Haubitz M, Hecker H, Mischak H, Fliser D. 2004. Proteomic patterns established with capillary electrophoresis and mass spectrometry for diagnostic purposes. *Kidney Int* **65:** (6) 2426.
White TA, Kell DB. 2004. Comparative genomic assessment of novel broad-spectrum targets for antibacterial drugs. *Comp Funct Genom* **5:** (4) 304.
Wreesmann VB, Sieczka EM, Socci ND, Hezel M, Belbin TJ, Childs G, Patel SG, Patel KN, Tallini G, Prystowsky M *et al*. 2004. Genome-wide profiling of papillary thyroid cancer identifies MUC1 as an independent prognostic marker. *Cancer Res* **64:** (11) 3780.
Yang ECC, Guo JZ, Diehl G, DeSouza L, Rodrigues MJ, Romaschin AD, Colgan TJ, Siu KWM. 2004. Protein expression profiling of endometrial malignancies reveals a new tumor marker: Chaperonin 10. *J Proteome Res* **3:** (3) 636.
Yang JW, Czech T, Lubec G. 2004. Proteomic profiling of human hippocampus. *Electrophoresis* **25:** (7-8) 1169.
Yao JJ, Chiba T, Sakai J, Hirose K, Yamamoto M, Hada A, Kuramoto K, Higuchi K, Mori M. 2004. Mouse testis transcriptome revealed using serial analysis of gene expression. *Mamm Genome* **15:** (6) 433.
Yeh W, Hanekamp T, Tsoka S, Karp PD, Altman RB. 2004. Computational analysis of *Plasmodium falciparum* metabolism: Organizing genomic information to facilitate drug discovery. *Genome Res* **14:** (5) 917.
Yu LR, Conrads TP, Uo T, Issaq HJ, Morrison RS, Veenstra TD. 2004. Evaluation of the acid-cleavable isotope-coded affinity tag reagents: Application to camptothecin-treated cortical neurons. *J Proteome Res* **3:** (3) 469.
Yuan Q, An J, Liu DG, Sun L, Ge YZ, Huang YL, Xu GJ, Zhao FK. 2004. Proteomic analysis of differential protein expression in a human hepatoma revertant cell line by using an improved two-dimensional electrophoresis procedure combined with matrix assisted laser desorption/ionization-time of flight-mass spectrometry. *Electrophoresis* **25:** (7-8) 1160.
Zang L, Toy DP, Hancock WS, Sgroi DC, Karger BL. 2004. Proteomic analysis of ductal carcinoma of the breast using laser capture microdissection, LC-MS, and $^{16}O/^{18}O$ isotopic labeling. *J Proteome Res* **3:** (3) 604.
Zhang GL, Wang GZ, Wang SY, Li QF, Ouyang AL, Peng XX. 2004. Applying proteomic methodologies to analyze the effect of hexamethylene bisacetamide (HMBA) on proliferation and differentiation of human gastric carcinoma BGC-823 cells. *Int J Biochem Cell Biol* **36:** (8) 1613.
Zhao ZJ, Raftery MJ, Niu XM, Daja MM, Russell PJ. 2004. Application of in-gel protease assay in a biological sample: Characterization and identification of urokinase-type plasminogen activator (uPA) in secreted proteins from a prostate cancer cell line PC-3. *Electrophoresis* **25:** (7-8) 1142.
Zhou L, Huang LQ, Beuerman RW, Grigg ME, Li SFY, Chew FT, Ang L, Stern ME, Tan D. 2004. Proteomic analysis of human tears: Defensin expression after ocular surface surgery. *J Proteome Res* **3:** (3) 410.
Zhou M, Lucas DA, Chan KC, Issaq HJ, Petricoin EF, Liotta LA, Veenstra TD, Conrads TR. 2004. An investigation into the human serum "interactome". *Electrophoresis* **25:** (9) 1289.

### 8 EST, cDNA and other clone resources

Alkharouf N, Khan R, Matthews B. 2004. Analysis of expressed sequence tags from roots of resistant soybean infected by the soybean cyst nematode. *Genome* **47:** (2) 380.
Beisel KW, Shiraki T, Morris KA, Pompeia C, Kachar B, Arakawa T, Bono H, Kawai J, Hayashizaki Y, Carninci P. 2004. Identification of unique transcripts from a mouse full-length, subtracted inner ear cDNA library. *Genomics* **83:** (6) 1012.
Chevreux B, Pfisterer T, Drescher B, Driesel AJ, Muller WEG, Wetter T, Suhai S. 2004. Using the miraEST assembler for reliable and automated mRNA transcript assembly and SNP detection in sequenced ESTs. *Genome Res* **14:** (6) 1147.
Cho SK, Ok SH, Jeung JU, Shim KS, Jung KW, You MK, Kang KH, Chung YS, Choi HC, Moon HP *et al*. 2004. Comparative analysis of 5,211 leaf ESTs of wild rice (*Oryza minuta*). *Plant Cell Rep* **22:** (11) 839.
Faix PH, Burg MA, Gonzales M, Ravey EP, Baird A, Larocca D. 2004. Phage display of cDNA libraries: Enrichment of cDNA expression using open reading frame selection. *Biotechniques* **36:** (6) 1018.
Handley D, Serban N, Peters D, O'Doherty R, Field M, Wasserman L, Spirtes P, Scheines R, Glymour C. 2004. Evidence of systematic expressed sequence tag IMAGE clone cross-hybridization on cDNA microarrays. *Genomics* **83:** (6) 1169.
Lee SH, Chae KS, Sohn DH. 2004. Identification of expressed sequence tags of genes expressed highly in the activated hepatic stellate cell. *Arch Pharm Research* **27:** (4) 422.
Parrish JR, Limjindaporn T, Hines JA, Liu JY, Liu GZ, Finley RL. 2004. High-throughput cloning of *Campylobacter jejuni* ORFs by *in vivo* recombination in *Escherichia coli*. *J Proteome Res* **3:** (3) 582.
Yamaguchi N, Kamino K, Ueki T, Michibata H. 2004. Expressed sequence tag analysis of vanadocytes in a vanadium-rich Ascidian, *Ascidia sydneiensis* Samea. *Mar Biotechnol* **6:** (2) 165.

### 9 Functional genomics

Askree SH, Yehuda T, Smolikov S, Gurevich R, Hawk J, Coker C, Krauskopf A, Kupiec M, McEachern MJM. 2004. A genome-wide screen for *Saccharomyces cerevisiae* deletion mutants that affect telomere length. *Proc Natl Acad Sci U S A* **101:** (23) 8658.
Berardini TZ, Mundodi S, Reiser L, Huala E, Garcia-Hernandez M, Zhang PF, Mueller LA, Yoon J, Doyle A, Lander G *et al*. 2004. Functional annotation of the *Arabidopsis* genome using controlled vocabularies. *Plant Physiol* **135:** (2) 745.
Bult C, Kibbe WA, Snoddy J, Vitaterna M, Swanson D, Pretel S, Li YX, Hohman MM, Rinchik E, Takahashi JS *et al*. 2004. A genome end-game: Understanding gene function in the nervous system. *Nat Neurosci* **7:** (5) 484.
Carrillo CD, Taboada E, Nash JHE, Lanthier P, Kelly J, Lau PC, Verhulp R, Mykytczuk O, Sy J, Findlay WA *et al*. 2004. Genome-wide expression analyses of *Campylobacter jejuni* NCTC11168 reveals coordinate regulation of motility and virulence by flhA. *J Biol Chem* **279:** (19) 20327.

Copyright © 2005 John Wiley & Sons, Ltd. 

Johnson MR, Montero CI, Conners SB, Shockley KR, Pysz MA, Kelly RM. 2004. Functional genomics-based studies of the microbial ecology of hyperthermophilic micro-organisms. *Biochem Soc Trans* **32:** (2) 188.

Semon M, Duret L. 2004. Evidence that functional transcription units cover at least half of the human genome. *Trends Genet* **20:** (5) 229.

Silva JM, Mizuno H, Brady A, Lucito R, Hannon GJ. 2004. RNA interference microarrays: High-throughput loss-of-function genetics in mammalian cells. *Proc Natl Acad Sci U S A* **101:** (17) 6548.

Skarnes WC, Von Melchner H, Wurst W, Hicks G, Nord AS, Cox T, Young SG, Ruiz P, Soriano P, Tessier-Lavigne M *et al*. 2004. A public gene trap resource for mouse functional genomics (Letter). *Nat Genet* **36:** (6) 543.

Smith S, Hwang JY, Banerjee S, Majeed A, Gupta A, Myung K. 2004. Mutator genes for suppression of gross chromosomal rearrangements identified by a genome-wide screening in *Saccharomyces cerevisiae*. *Proc Natl Acad Sci U S A* **101:** (24) 9039.

Van der Linden AM, Plasterk RHA. 2004. Shotgun cloning of transposon insertions in the genome of *Caenorhabditis elegans*. *Comp Funct Genom* **5:** (3) 225.

Vilella F, Alves R, Rodriguez-Manzaneque MT, Belli G, Swaminathan S, Sunnerhagen P, Herrero E. 2004. Evolution and cellular function of monothiol glutaredoxins: Involvement in iron-sulphur cluster assembly. *Comp Funct Genom* **5:** (4) 328.

### I0 Transcriptomics

Alic N, Felder T, Temple MD, Gloeckner C, Higgins VJ, Briza P, Dawes IW. 2004. Genome-wide transcriptional responses to a lipid hydroperoxide: Adaptation occurs without induction of oxidant defenses. *Free Radic Biol Med* **37:** (1) 23.

Asyali MH, Shoukri MM, Demirkaya O, Khabar KSA. 2004. Assessment of reliability of microarray data and estimation of signal thresholds using mixture modeling. *Nucleic Acids Res* **32:** (8) 2323.

Bolduc C, Larose M, Lafond N, Yoshioka M, Rodrigue MA, Morissette J, Labrie C, Raymond V, St Amand J. 2004. Adipose tissue transcriptome by serial analysis of gene expression. *Obes Res* **12:** (5) 750.

Duncan R. 2004. DNA microarray analysis of protozoan parasite gene expression: Outcomes correlate with mechanisms of regulation. *Trends Parasitol* **20:** (5) 211.

Espanhol AR, Cardoso RS, Junta CM, Victorero G, Loriod B, Nguyen C, Passos GAS. 2004. Large scale gene expression analysis of CBA/J mouse strain fetal thymus using cDNA-array hybridizations. *Mol Cell Biochem* **260:** (1) 65.

Fujita K, Matsuyama A, Kobayashi Y, Iwahashi H. 2004. Comprehensive gene expression analysis of the response to straight-chain alcohols in *Saccharomyces cerevisiae* using cDNA microarray. *J Appl Microbiol* **97:** (1) 57.

Galante PAF, Sakabe NJ, Kirschbaum-Slager N, De Souza SJ. 2004. Detection and evaluation of intron retention events in the human transcriptome. *RNA* **10:** (5) 757.

Gechev TS, Gadjev IZ, Hille J. 2004. An extensive microarray analysis of AAL-toxin-induced cell death in *Arabidopsis thaliana* brings new insights into the complexity of programmed cell death in plants. *Cell Mol Life Sci* **61:** (10) 1185.

Gosset G, Zhang ZG, Nayyar SN, Cuevas WA, Saier MH. 2004. Transcriptome analysis of Crp-dependent catabolite control of gene expression in *Escherichia coli*. *J Bacteriol* **186:** (11) 3516.

Guo Y, Cai Z, Gan S. 2004. Transcriptome of *Arabidopsis* leaf senescence. *Plant Cell Environ* **27:** (5) 521.

Han JS, Szak ST, Boeke JD. 2004. Transcriptional disruption by the L1 retrotransposon and implications for mammalian transcriptomes. *Nature* **429:** (6989) 268.

Ichimura T, Kubota H, Goma T, Mizushima N, Ohsumi Y, Iwago M, Kakiuchi K, Shekhar HU, Shinkawa T, Taoka M, Ito T, Isobe T. 2004. Transcriptomic and proteomic analysis of a 14-3-3 gene-deficient yeast. *Biochemistry* **43:** (20) 6149.

Jarvinen AK, Hautaniemi S, Edgren H, Auvinen P, Saarela J, Kallioniemi OP, Monni O. 2004. Are data from different gene expression microarray platforms comparable? *Genomics* **83:** (6) 1164.

Jasinska A, Krzyzosiak WJ. 2004. Repetitive sequences that shape the human transcriptome. *FEBS Lett* **567:** (1) 136.

Kashima S, Roberto PG, Soares AM, Astolfi-Filho S, Pereira JO, Giuliati S, Faria M, Xavier MAS, Fontes MRM, Giglio JR *et al*. 2004. Analysis of *Bothrops jararacussu* venomous gland transcriptome focusing on structural and functional aspects: 1 - Gene expression profile of highly expressed phospholipases $A_2$. *Biochimie* **86:** (3) 211.

Ko JH, Han KH, Park S, Yang JM. 2004. Plant body weight-induced secondary growth in *Arabidopsis* and its transcription phenotype revealed by whole-transcriptome profiling. *Plant Physiol* **135:** (2) 1069.

Lee HK, Hsu AK, Sajdak J, Qin J, Pavlidis P. 2004. Coexpression analysis of human genes across many microarray data sets. *Genome Res* **14:** (6) 1085.

Lloyd JC, Zakhleniuk OV. 2004. Responses of primary and secondary metabolism to sugar accumulation revealed by microarray expression analysis of the *Arabidopsis* mutant, pho3. *J Exp Bot* **55:** (400) 1221.

Mera N, Aoyagi H, Nakasono S, Iwasaki K, Saiki H, Tanaka H. 2004. Analysis of gene expression in yeast protoplasts using DNA microarrays and their application for efficient production of invertase and α-glucosidase. *J Biosci Bioeng* **97:** (3) 169.

Niehus E, Gressmann H, Ye F, Schlapbach R, Dehio M, Dehio C, Stack A, Meyer TF, Suerbaum S, Josenhans C. 2004. Genome-wide analysis of transcriptional hierarchy and feedback regulation in the flagellar system of *Helicobacter pylori*. *Mol Microbiol* **52:** (4) 947.

Obayashi T, Okegawa T, Sasaki-Sekimoto Y, Shimada H, Masuda T, Asamizu E, Nakamura Y, Shibata D, Tabata S, Takamiya KI *et al*. 2004. Distinctive features of plant organs characterized by global analysis of gene expression in *Arabidopsis*. *DNA Res* **11:** (1) 11.

Palmer E, Freeman T. 2004. Investigation into the use of C- and N-terminal GFP fusion proteins for subcellular localization studies using reverse transfection microarrays. *Comp Funct Genom* **5:** (4) 342.

Pedra JHF, McIntyre LM, Scharf ME, Pittendrigh BR. 2004. Genome wide transcription profile of field- and laboratory-selected dichlorodiphenyltrichloroethane (DDT)-resistant *Drosophila*. *Proc Natl Acad Sci U S A* **101:** (18) 7034.

Portman DS, Emmons SW. 2004. Identification of *C. elegans* sensory ray genes using whole-genome expression profiling. *Dev Biol* **270:** (2) 499.

Pysz MA, Ward DE, Shockley KR, Montero CI, Conners SB, Johnson MR, Kelly RM. 2004. Transcriptional analysis of dynamic heat-shock response by the hyperthermophilic bacterium *Thermotoga maritima*. *Extremophiles* **8:** (3) 209.

Rehwinkel J, Herold A, Gari K, Kocher T, Rode M, Ciccarelli FL, Wilm M, Izaurralde E. 2004. Genome-wide analysis of mRNAs regulated by the THO complex in *Drosophila melanogaster*. *Nat Struct Mol Biol* **11:** (6) 558.

Ribeiro JMC, Charlab R, Pham VM, Garfield M, Valenzuela JG. 2004. An insight into the salivary transcriptome and proteome of the adult female mosquito *Culex pipiens quinquefasciatus*. *Insect Biochem Mol Biol* **34:** (6) 543.

Riva A, Delorme MO, Chevalier T, Guilhot N, Henaut C, Henaut A. 2004. The difficult interpretation of transcriptome data: The case of the GATC regulatory network. *Comput Biol Chem* **28:** (2) 109.

Rudd S, Frisch M, Grote K, Meyers BC, Mayer K, Werner T. 2004. Genome-wide *in silico* mapping of scaffold/matrix attachment regions in *Arabidopsis* suggests correlation of intragenic scaffold/matrix attachment regions with gene expression. *Plant Physiol* **135:** (2) 715.

Ruse CI, Tan FL, Kinter M, Bond M. 2004. Integrated analysis of the human cardiac transcriptome, proteome and phosphoproteome. *Proteomics* **4:** (5) 1505.

Spira A, Schembri F, Beane J, Shah V, Liu G, Brody JS. 2004. Impact of cigarette smoke on the normal airway transcriptome. *Chest* **125:** (Suppl 5) 115S.

Taib Z. 2004. Statistical analysis of oligonucleotide microarray data. *C R Biol* **327:** (3) 175.

Taylor TB, Nambiar PR, Raja R, Cheung E, Rosenberg DW, Anderegg B. 2004. Microgenomics: Identification of new expression profiles *via* small and single-cell sample analyses. *Cytometry* **59A:** (2) 254.

Tucker CL, Fields S. 2004. Quantitative genome-wide analysis of yeast deletion strain sensitivities to oxidative and chemical stress. *Comp Funct Genom* **5:** (3) 216.

Vaughan A, Mahony JO, Eijsink VGH, O'Connell-Motherway M, Van Sinderen D. 2004. Transcriptional analysis of bacteriocin production by malt isolate *Lactobacillus sakei* 5. *FEMS Microbiol Lett* **235:** (2) 377.

Copyright © 2005 John Wiley & Sons, Ltd. 

### II Proteomics

Abdolzade-Bavil A, Hayes S, Goretzki L, Kroger M, Anders J, Hendriks R. 2004. Convenient and versatile subcellular extraction procedure, that facilitates classical protein expression profiling and functional protein analysis. *Proteomics* **4:** (5) 1397.

Archambault V, Chang EJ, Drapkin BJ, Cross FR, Chait BT, Rout MP. 2004. Targeted proteomic study of the cyclin-Cdk module. *Mol Cell* **14:** (6) 699.

Arevalo-Ferro C, Buschmann J, Reil G, Gorg A, Wiehlmann L, Tummler B, Eberl L, Riedel K. 2004. Proteome analysis of intraclonal diversity of two *Pseudomonas aeruginosa* TB clone isolates. *Proteomics* **4:** (5) 1241.

Bigelow HR, Petrey DS, Liu J, Przybylski D, Rost B. 2004. Predicting transmembrane β-barrels in proteomes. *Nucleic Acids Res* **32:** (8) 2566.

Blom N, Sicheritz-Ponten T, Gupta R, Gammeltoft S, Brunak S. 2004. Prediction of post-translational glycosylation and phosphorylation of proteins from the amino acid sequence. *Proteomics* **4:** (6) 1633.

Blonder J, Hale ML, Lucas DA, Schaefer CF, Yu LR, Conrads TR, Issaq HJ, Stiles BG, Veenstra TD. 2004. Proteomic analysis of detergent-resistant membrane rafts. *Electrophoresis* **25:** (9) 1307.

Blonder J, Goshe MB, Xiao WZ, Camp DG, Wingerd M, Davis RW, Smith RD. 2004. Global analysis of the membrane subproteome of *Pseudomonas aeruginosa* using liquid chromatography-tandem mass spectrometry. *J Proteome Res* **3:** (3) 434.

Blume C, Lindner B, Becker WM, Petersen A. 2004. Microheterogeneity of the major grass group 6 allergen Phl p 6: Analysis by mass spectrometry. *Proteomics* **4:** (5) 1366.

Bologna G, Yvon C, Duvaud S, Veuthey AL. 2004. *N*-Terminal myristoylation predictions by ensembles of neural networks. *Proteomics* **4:** (6) 1626.

Bouley J, Chambon C, Picard B. 2004. Mapping of bovine skeletal muscle proteins using two-dimensional gel electrophoresis and mass spectrometry. *Proteomics* **4:** (6) 1811.

Brokx SJ, Ellison M, Locke T, Bottorff D, Frost L, Weiner JH. 2004. Genome-wide analysis of lipoprotein expression in *Escherichia coli* MG1655. *J Bacteriol* **186:** (10) 3254.

Carugo O, Franzot G. 2004. Prediction of protein-protein interactions based on surface patch comparison. *Proteomics* **4:** (6) 1727.

Chao CC, Chelius D, Zhang T, Daggle L, Ching WM. 2004. Proteome analysis of Madrid E strain of *Rickettsia prowazekii*. *Proteomics* **4:** (5) 1280.

Chevalier F, Martin O, Rofidal V, Devauchelle AD, Barteau S, Sommerer N, Rossignol M. 2004. Proteomic investigation of natural variation between *Arabidopsis* ecotypes. *Proteomics* **4:** (5) 1372.

Coelho A, Santos EDO, Faria MLDH, De Carvalho DP, Soares MR, Von Kruger WMA, Bisch PM. 2004. A proteome reference map for *Vibrio cholerae* El Tor. *Proteomics* **4:** (5) 1491.

Conrads KA, Yu LR, Lucas DA, Zhou M, Chan KC, Simpson KA, Schaefer CF, Issaq HJ, Veenstra TD, Beck GR *et al*. 2004. Quantitative proteomic analysis of inorganic phosphate-induced murine MC3T3-E1 osteoblast cells. *Electrophoresis* **25:** (9) 1342.

Drews O, Reil G, Parlar H, Gorg A. 2004. Setting up standards and a reference map for the alkaline proteome of the Gram-positive bacterium *Lactococcus lactis*. *Proteomics* **4:** (5) 1293.

Dumas-Gaudot E, Valot B, Bestel-Corre G, Recorbet G, St-Arnaud M, Fontaine B, Dieu M, Raes M, Saravanan RS, Gianinazzi S. 2004. Proteomics as a way to identify extra-radicular fungal proteins from *Glomus* intraradices - RiT-DNA carrot root mycorrhizas. *FEMS Microbiol Ecol* **48:** (3) 401.

Dworzanski JP, Snyder AP, Chen R, Zhang HY, Wishart D, Li L. 2004. Identification of bacteria using tandem mass spectrometry combined with a proteome database and statistical scoring. *Anal Chem* **76:** (8) 2355.

Eisenhaber B, Eisenhaber F, Maurer-Stroh S, Neuberger G. 2004. Prediction of sequence signals for lipid post-translational modifications: Insights from case studies. *Proteomics* **4:** (6) 1614.

Fly BG, Wuster W. 2004. Assembling an arsenal: Origin and evolution of the snake venom proteome inferred from phylogenetic analysis of toxin sequences. *Mol Biol Evol* **21:** (5) 870.

Gasnier C, Lugardon K, Ruh O, Strub JM, Aunis D, Metz-Boutigue MH. 2004. Characterization and location of post-translational modifications on chromogranin B from bovine adrenal medullary chromaffin granules. *Proteomics* **4:** (6) 1789.

Giordano A, Russo C, Raia CA, Kuznetsova IM, Stepanenko OV, Turoverov KK. 2004. Highly UV-absorbing complex in selenomethionine-substituted alcohol dehydrogenase from *Sulfolobus solfataricus*. *J Proteome Res* **3:** (3) 613.

Gonnet P, Rudd KE, Lisacek F. 2004. Fine-tuning the prediction of sequences cleaved by signal peptidase II: A curated set of proven and predicted lipoproteins of *Escherichia coli* K-12. *Proteomics* **4:** (6) 1597.

Hassel S, Eichner A, Yakymovych M, Hellman U, Knaus P, Soucheinytskyi S. 2004. Proteins associated with type II bone morphogenetic protein receptor (BMPR-II) and identified by two-dimensional gel electrophoresis and mass spectrometry. *Proteomics* **4:** (5) 1346.

Heintz D, Wurtz V, High AA, Van Dorsselaer A, Reski R, Sarnighausen E. 2004. An efficient protocol for the identification of protein phosphorylation in a seedless plant, sensitive enough to detect members of signalling cascades. *Electrophoresis* **25:** (7-8) 1149.

Hjerrild M, Stensballe A, Rasmussen TE, Kofoed CB, Blom N, Sicheritz-Ponten T, Larsen MR, Brunak S, Jensen ON, Gammeltoft S. 2004. Identification of phosphorylation sites in protein kinase A substrates using artificial neural networks and mass spectrometry. *J Proteome Res* **3:** (3) 426.

Hunsucker SW, Klage K, Slaughter SM, Potts M, Helm RF. 2004. A preliminary investigation of the *Nostoc punctiforme* proteome. *Biochem Biophys Res Commun* **317:** (4) 1121.

Imanishi S, Harada K. 2004. Proteomics approach on microcystin binding proteins in mouse liver for investigation of microcystin toxicity. *Toxicon* **43:** (6) 651.

Jalili PR, Gheyi T, Dass C. 2004. Proteome analysis in bovine adrenal medulla using matrix-assisted laser desorption/ionization mass spectrometry (Letter). *Rapid Commun Mass Spectrom* **18:** (8) 914.

Koga H, Shimada K, Hara Y, Nagan M, Kohga H, Yokoyama R, Kimura Y, Yuasa S, Magae J, Inamoto S *et al*. 2004. A comprehensive approach for establishment of the platform to analyze functions of KIAA proteins: Generation and evaluation of anti-mKIAA antibodies. *Proteomics* **4:** (5) 1412.

Lai EM, Nair U, Phadke ND, Maddock JR. 2004. Proteomic screening and identification of differentially distributed membrane proteins in *Escherichia coli*. *Mol Microbiol* **52:** (4) 1029.

Laukel M, Rossignol M, Borderies G, Volker U, Vorholt JA. 2004. Comparison of the proteome of *Methylobacterium extorquens* AM1 grown under methylotrophic and nonmethylotrophic conditions. *Proteomics* **4:** (5) 1247.

Lee S, Lee EJ, Yang EJ, Lee JE, Park AR, Song WH, Park OK. 2004. Proteomic identification of annexins, calcium-dependent membrane binding proteins that mediate osmotic stress and abscisic acid signal transduction in *Arabidopsis*. *Plant Cell* **16:** (6) 1378.

Leonoudakis D, Conti LR, Anderson S, Radeke CM, McGuire LMM, Adams ME, Froehner SC, Yates JR, Vandenberg CA. 2004. Protein trafficking and anchoring complexes revealed by proteomic analysis of inward rectifier potassium channel (Kir2.x)-associated proteins. *J Biol Chem* **279:** (21) 22331.

Li TW, Evdokimov E, Shen RF, Chao CC, Tekle E, Wang T, Stadtman ER, Yang DCH, Chock PB. 2004. Sumoylation of heterogeneous nuclear ribonucleoproteins, zinc finger proteins, and nuclear pore complex proteins: A proteomic analysis. *Proc Natl Acad Sci U S A* **101:** (23) 8551.

Liberatori S, Canas B, Tani C, Bini L, Buonocore G, Godovac-Zimmermann J, Mishra OP, Delivoria-Papadopoulos M, Bracci R, Pallini V. 2004. Proteomic approach to the identification of voltage-dependent anion channel protein isoforms in guinea pig brain synaptosomes. *Proteomics* **4:** (5) 1335.

Marrero J, Gonzalez LJ, Sanchez A, Ayala M, Paz-Lago D, Gonzalez W, Fallarero A, Castellanos-Serra L, Coto O. 2004. Effect of high concentration of Co(II) on *Enterobacter liquefaciens* strain C-1: A bacterium highly resistant to heavy metals with an unknown genome. *Proteomics* **4:** (5) 1265.

Muth E, Driscoll WJ, Smalstig A, Goping G, Mueller GP. 2004. Proteomic analysis of rat atrial secretory granules: A platform for testable hypotheses. *Biochim Biophys Acta* **1699:** (1-2) 263.

Copyright © 2005 John Wiley & Sons, Ltd. 

Nawarak J, Phutrakul S, Chen ST. 2004. Analysis of lectin-bound glycoproteins in snake venom from the Elapidae and Viperidae families. *J Proteome Res* **3:** (3) 383.

Nylund R, Leszczynski D. 2004. Proteomics analysis of human endothelial cell line EA.hy926 after exposure to GSM 900 radiation. *Proteomics* **4:** (5) 1359.

Ostrowski M, Fegatella F, Wasinger V, Guilhaus M, Corthals GL, Cavicchioli R. 2004. Cross-species identification of proteins from proteome profiles of the marine oligotrophic ultramicrobacterium, *Sphingopyxis alaskensis*. *Proteomics* **4:** (6) 1779.

Pejovic V, Soskic V, Pan W, Kastin AJ. 2004. Brain proteome of mice lacking the receptors for tumor necrosis factor α. *Proteomics* **4:** (5) 1461.

Peng JM, Kim MJ, Cheng DM, Duong DM, Gygi SP, Sheng M. 2004. Semiquantitative proteomic analysis of rat forebrain postsynaptic density fractions by mass spectrometry. *J Biol Chem* **279:** (20) 21003.

Predel R, Wegener C, Russell WK, Tichy SE, Russell DH, Nachman RJ. 2004. Peptidomics of CNS-associated neurohemal systems of adult *Drosophila melanogaster*: A mass spectrometric survey of peptides from individual flies. *J Comp Neurol* **474:** (3) 379.

Reczko M, Hatzigeorgiou A. 2004. Prediction of the subcellular localization of eukaryotic proteins using sequence signals and composition. *Proteomics* **4:** (6) 1591.

Schmidt I, Steenbakkers PJM, Op den Camp HJM, Schmidt K, Jetten MSM. 2004. Physiologic and proteomic evidence for a role of nitric oxide in biofilm formation by *Nitrosomonas europaea* and other ammonia oxidizers. *J Bacteriol* **186:** (9) 2781.

Sharma R, Komatsu S, Noda H. 2004. Proteomic analysis of brown planthopper: Application to the study of carbamate toxicity. *Insect Biochem Mol Biol* **34:** (5) 425.

Shi XD, Belton RJ, Burkin HR, Vieira AP, Miller DJ. 2004. A proteomic approach to identify phosphoproteins encoded by cDNA libraries. *Anal Biochem* **329:** (2) 289.

Shimazaki Y, Sugawara Y, Manabe T. 2004. Nondenaturing two-dimensional electrophoresis enzyme profile involving activity and sequence structure of cytosol proteins from mouse liver. *Proteomics* **4:** (5) 1406.

Smith AP, DeRidder BP, Guo WJ, Seeley EH, Regnier FE, Goldsbrough PB. 2004. Proteomic analysis of *Arabidopsis* glutathione *S*-transferases from benoxacor- and copper-treated seedlings. *J Biol Chem* **279:** (25) 26098.

Southan C. 2004. Has the yo-yo stopped? - An assessment of human protein-coding gene number. *Proteomics* **4:** (6) 1712.

Sun Q, Emanuelsson O, Van Wijk KJ. 2004. Analysis of curated and predicted plastid subproteomes of *Arabidopsis*, subcellular compartmentalization leads to distinctive proteome properties. *Plant Physiol* **135:** (2) 723.

Tam EM, Morrison CJ, Wu YI, Stack MS, Overall CM. 2004. Membrane protease proteomics: Isotope-coded affinity tag MS identification of undescribed MT1-matrix metalloproteinase substrates. *Proc Natl Acad Sci U S A* **101:** (18) 6917.

Taylor TJ, Knipe DM. 2004. Proteomics of herpes simplex virus replication compartments: Association of cellular DNA replication, repair, recombination, and chromatin remodeling proteins with ICP8. *J Virol* **78:** (11) 5856.

Vilain S, Cosette P, Hubert M, Lange C, Junter GA, Jouenne T. 2004. Comparative proteomic analysis of planktonic and immobilized *Pseudomonas aeruginosa* cells: A multivariate statistical approach. *Anal Biochem* **329:** (1) 120.

Voigt B, Schweder T, Becher R, Ehrenreich A, Gottschalk G, Feesche J, Maurer KH, Hecker M. 2004. A proteomic view of cell physiology of *Bacillus licheniformis*. *Proteomics* **4:** (5) 1465.

Wrzeszczynski KO, Rost B. 2004. Annotating proteins from endoplasmic reticulum and Golgi apparatus in eukaryotic proteomes. *Cell Mol Life Sci* **61:** (11) 1341.

Wu CC, MacCoss MJ, Mardones G, Finnigan C, Mogelsvang S, Yates JR, Howell KE. 2004. Organellar proteomics reveals Golgi arginine dimethylation. *Mol Biol Cell* **15:** (6) 2907.

Yoshikuni M, Sagegami R, Nagahama Y. 2003. Proteome analysis: A new approach to identify key proteins involved in acquisition of maturational competence and oocyte maturation of medaka oocytes. *Fish Physiol Biochem* **28:** (1-4) 379.

Zeng R, Ruan HQ, Jiang XS, Zhou H, Shi L, Zhang L, Sheng QH, Tu QA, Xia QC, Wu JR. 2004. Proteomic analysis of SARS associated coronavirus using two-dimensional liquid chromatography mass spectrometry and one-dimensional sodium dodecyl sulfate-polyacrylamide gel electrophoresis followed by mass spectrometric analysis. *J Proteome Res* **3:** (3) 549.

Zhu WH, Reich CI, Olsen GJ, Giometti CS, Yates JR. 2004. Shotgun proteomics of *Methanococcus jannaschii* and insights into methanogenesis. *J Proteome Res* **3:** (3) 538.

Zolla L, Rinalducci S, Timperio AM, Huber CG, Righetti PG. 2004. Intact mass measurements for unequivocal identification of hydrophobic photosynthetic photosystems I and II antenna proteins. *Electrophoresis* **25:** (9) 1353.

### I2 Protein structural genomics

Canaves JM. 2004. Predicted role for the archease protein family based on structural and sequence analysis of TM1083 and MTH1598, two proteins structurally characterized through structural genomics efforts. *Proteins* **56:** (1) 19.

Kuznetsova IM, Turoverov KK, Uversky VN. 2004. Use of the phase diagram method to analyze the protein unfolding-refolding reactions: Fishing out the “invisible” intermediates. *J Proteome Res* **3:** (3) 485.

Martelli PL, Fariselli P, Casadio R. 2004. Prediction of disulfide-bonded cysteines in proteomes with a hidden neural network. *Proteomics* **4:** (6) 1665.

### I3 Metabolomics

Len MCL, Harty DWS, Jacques NA. 2004. Proteome analysis of *Streptococcus mutans* metabolic phenotype during acid tolerance. *Microbiology* **150:** (5) 1353.

Lenz EM, Bright J, Knight R, Wilson ID, Major H. 2004. Cyclosporin A-induced changes in endogenous metabolites in rat urine: A metabonomic investigation using high field $^{1}$H NMR spectroscopy, HPLC-TOF/MS and chemometrics. *J Pharm Biomed Anal* **35:** (3) 599.

Mayr M, Siow R, Chung YL, Mayr U, Griffiths JR, Xu QB. 2004. Proteomic and metabolomic analysis of vascular smooth muscle cells - Role of PKCδ. *Circ Res* **94:** (10) E87.

Svetic RE, MacCluer CR, Buckley CO, Smythe KL, Jackson JH. 2004. A metabolic force for gene clustering. *Bull Math Biol* **66:** (3) 559.

Zaslaver A, Mayo AE, Rosenberg R, Bashkin P, Sberro H, Tsalyuk M, Surette MG, Alon U. 2004. Just-in-time transcription program in metabolic pathways. *Nat Genet* **36:** (5) 486.

### I4 Genomic approaches to development

Afrakhte M, Schultheiss TM. 2004. Construction and analysis of a subtracted library and microarray of cDNAs expressed specifically in chicken heart progenitor cells. *Dev Dyn* **230:** (2) 290.

Akopyants NS, Matlib RS, Bukanova EN, Smeds MR, Brownstein BH, Stormo GD, Beverley SM. 2004. Expression profiling using random genomic DNA microarrays identifies differentially expressed genes associated with three major developmental stages of the protozoan parasite *Leishmania major*. *Mol Biochem Parasitol* **136:** (1) 71.

Almeida R, Gilmartin BJ, McCann SH, Norrish A, Ivens AC, Lawson D, Levick MP, Smith DF, Dyall SD, Vetrie D *et al*. 2004. Expression profiling of the *Leishmania* life cycle: cDNA arrays identify developmentally regulated genes present but not annotated in the genome. *Mol Biochem Parasitol* **136:** (1) 87.

Arbeitman MN, Fleming AA, Siegal ML, Null BH, Baker BS. 2004. A genomic analysis of *Drosophila* somatic sexual differentiation and its regulation. *Development* **131:** (9) 2007.

Brandenberger R, Wei H, Zhang S, Lei S, Murage J, Fisk GJ, Li Y, Xu CH, Fang R, Guegler K *et al*. 2004. Transcriptome characterization elucidates signaling networks that control human ES cell growth and differentiation. *Nat Biotechnol* **22:** (6) 707.

Choi KL, Wang Y, Tse CA, Lam KSL, Cooper GJS, Xu AM. 2004. Proteomic analysis of adipocyte differentiation: Evidence that α2 macroglobulin is involved in the adipose conversion of 3T3 L1 preadipocytes. *Proteomics* **4:** (6) 1840.

Copyright © 2005 John Wiley & Sons, Ltd. 

Evans CA, Tonge R, Blinco D, Pierce A, Shaw J, Lu YN, Hamzah HG, Gray A, Downes CP, Gaskell SJ *et al*. 2004. Comparative proteomics of primitive hematopoietic cell populations reveals differences in expression of proteins regulating motility. *Blood* **103:** (10) 3751.

Hu JB, Gray CA, Spencer TE. 2004. Gene expression profiling of neonatal mouse uterine development. *Biol Reprod* **70:** (6) 1870.

Jung YK, Jeong JH, Ryoo HM, Kim HN, Kim YJ, Park EK, Si HJ, Kim SY, Takigawa M, Lee BH *et al*. 2004. Gene expression profile of human chondrocyte HCS-2/8 cell line by EST sequencing analysis. *Gene* **330:** 85.

Larose M, St-Amand J, Yoshioka M, Belleau P, Morissette J, Labrie C, Raymond V, Labrie F. 2004. Transcriptome of mouse uterus by serial analysis of gene expression (SAGE): Comparison with skeletal muscle. *Mol Reprod Dev* **68:** (2) 142.

Lim CR, Fukakusa A, Matsubara K. 2004. Gene expression profiling of mouse postnatal cerebellar development using cDNA microarrays. *Gene* **333:** 3.

Mikheeva S, Barrier M, Little SA, Beyer R, Mikheev AM, Kerr MK, Mirkes PE. 2004. Alterations in gene expression induced in day-9 mouse embryos exposed to hyperthermia (HS) or 4-hydroperoxycyclophosphamide (4CP): Analysis using cDNA microarrays. *Toxicol Sci* **79:** (2) 345.

Morris JS, Davies CR, Griffiths MR, Page MJ, Bruce JA, Patel T, Herath A, Gusterson BA. 2004. Proteomic analysis of mouse mammary terminal end buds identifies axonal growth cone proteins. *Proteomics* **4:** (6) 1802.

Nirmalan N, Sims PFG, Hyde JE. 2004. Quantitative proteomics of the human malaria parasite *Plasmodium falciparum* and its application to studies of development and inhibition. *Mol Microbiol* **52:** (4) 1187.

Nugent PG, Karsani SA, Wait R, Tempero J, Smith DF. 2004. Proteomic analysis of *Leishmania mexicana* differentiation. *Mol Biochem Parasitol* **136:** (1) 51.

Paba J, Ricart CAO, Fontes W, Santana JM, Teixeira ARL, Marchese J, Williamson B, Hunt T, Karger BL, Sousa MV. 2004. Proteomic analysis of *Trypanosoma cruzi* developmental stages using isotope-coded affinity tag reagents. *J Proteome Res* **3:** (3) 517.

Pompeia C, Hurle B, Belyantseva IA, Noben-Trauth K, Beisel K, Gao J, Buchoff P, Wistow G, Kachar B. 2004. Gene expression profile of the mouse organ of corti at the onset of hearing. *Genomics* **83:** (6) 1000.

Tang MK, Kindler PM, Cai DQ, Chow PH, Li M, Lee KKH. 2004. Heart-type fatty acid binding proteins are upregulated during terminal differentiation of mouse cardiomyocytes, as revealed by proteomic analysis. *Cell Tissue Res* **316:** (3) 339.

### I5 Technological advances

Arnold RJ, Hrncirova P, Annaiah K, Novotny MV. 2004. Fast proteolytic digestion coupled with organelle enrichment for proteomic analysis of rat liver. *J Proteome Res* **3:** (3) 653.

Barnidge DR, Hall GD, Stocker JL, Muddiman DC. 2004. Evaluation of a cleavable stable isotope labeled synthetic peptide for absolute protein quantification using LC-MS/MS. *J Proteome Res* **3:** (3) 658.

Belov ME, Anderson GA, Wingerd MA, Udseth HR, Tang KQ, Prior DC, Swanson KR, Buschbach MA, Strittmatter EF, Moore RJ, Smith RD. 2004. An automated high performance capillary liquid chromatography-Fourier transform ion cyclotron resonance mass spectrometer for high-throughput proteomics. *J Am Soc Mass Spectrom* **15:** (2) 212.

Bonaldi T, Imhof A, Regula JT. 2004. A combination of different mass spectroscopic techniques for the analysis of dynamic changes of histone modifications. *Proteomics* **4:** (5) 1382.

Canaves JM, Morse A, West B. 2004. PCR primer selection tool optimized for high-throughput proteomics and structural genomics. *Biotechniques* **36:** (6) 1040.

Candiano G, Bruschi M, Musante L, Santucci L, Ghiggeri GM, Carnemolla B, Orecchia P, Zardi L, Righetti PG. 2004. Blue silver: A very sensitive colloidal Coomassie G-250 staining for proteome analysis. *Electrophoresis* **25:** (9) 1327.

Carvalho B, Ouwerkerk E, Meijer GA, Ylstra B. 2004. High resolution microarray comparative genomic hybridisation analysis using spotted oligonucleotides. *J Clin Pathol* **57:** (6) 644.

Choi JK, Chae HZ, Hwang SY, Choi HI, Jin LT, Yoo GS. 2004. Fast visible dye staining of proteins in one- and two-dimensional sodium dodecyl sulfate-polyacrylamide gels compatible with matrix assisted laser desorption/ionization-mass spectrometry. *Electrophoresis* **25:** (7-8) 1136.

Cooper JW, Gao J, Lee CS. 2004. Electronic gel protein transfer and identification using matrix-assisted laser desorption/ionization-mass spectrometry. *Electrophoresis* **25:** (9) 1379.

Denison C, Kodadek T. 2004. Toward a general chemical method for rapidly mapping multi-protein complexes. *J Proteome Res* **3:** (3) 417.

Dominique SR, Gruissem W, Baginsky S. 2004. Mass spectrometric identification of RNA binding proteins from dried EMSA gels. *J Proteome Res* **3:** (3) 662.

Fan JB, Yeakley JM, Bibikova M, Chudin E, Wickham E, Chen J, Doucet D, Rigault P, Zhang BH, Shen R *et al*. 2004. A versatile assay for high-throughput gene expression profiling on universal array matrices. *Genome Res* **14:** (5) 878.

Galperin MM, Traicoff JL, Ramesh A, Freebern WJ, Haggerty CM, Hartmann DP, Emmert-Buck MR, Gardner K, Knezevic V. 2004. Multimembrane dot-blotting: A cost-effective tool for proteome analysis. *Biotechniques* **36:** (6) 1046.

Gilbert L, Schiffmann S, Rubenwolf S, Jensen K, Mail T, Albrecht C, Lankenau A, Beste G, Blank K, Gaub HE *et al*. 2004. Double chip protein arrays using recombinant single-chain Fv antibody fragments. *Proteomics* **4:** (5) 1417.

Gires O, Munz M, Schaffrik M, Kieu C, Rauch J, Ahlemann M, Eberle D, Mack B, Wollenberg B, Lang S *et al*. 2004. Profile identification of disease-associated humoral antigens using AMIDA, a novel proteomics-based technology. *Cell Mol Life Sci* **61:** (10) 1198.

Gorreta F, Barzaghi D, Van Meter AJ, Chandhoke V, Del Giacco L. 2004. Development of a new reference standard for microarray experiments. *Biotechniques* **36:** (6) 1002.

Hagglund P, Bunkenborg J, Elortza F, Jensen ON, Roepstorff P. 2004. A new strategy for identification of N-glycosylated proteins and unambiguous assignment of their glycosylation sites using HILIC enrichment and partial deglycosylation. *J Proteome Res* **3:** (3) 556.

Hagman C, Ramstrom M, Hakansson P, Bergquist J. 2004. Quantitative analysis of tryptic protein mixtures using electrospray ionization Fourier transform ion cyclotron resonance mass spectrometry. *J Proteome Res* **3:** (3) 587.

Hanaki K, Ohka S, Yamamoto K, Nomoto A, Yoshikura H. 2004. Oligo(dA-dT)-dependent signal amplification for the detection of proteins in cells. *Biotechniques* **36:** (5) 856.

Harper RG, Workman SR, Schuetzner S, Timperman AT, Sutton JN. 2004. Low-molecular-weight human serum proteome using ultrafiltration, isoelectric focusing, and mass spectrometry. *Electrophoresis* **25:** (9) 1299.

Jorgensen CS, Jagd M, Sorensen BK, McGuire J, Barkholt V, Hojrup P, Houen G. 2004. Efficacy and compatibility with mass spectrometry of methods for elution of proteins from sodium dodecyl sulfate-polyacrylamide gels and polyvinyldifluoride membranes. *Anal Biochem* **330:** (1) 87.

Joshi H, Harrison M, Schulz B, Cooper C, Packer N, Karlsson N. 2004. Development of a mass fingerprinting tool for automated interpretation of oligosaccharide fragmentation data. *Proteomics* **4:** (6) 1650.

Karp NA, Kreil DP, Lilley KS. 2004. Determining a significant change in protein expression with DeCyder™ during a pair-wise comparison using two-dimensional difference gel electrophoresis. *Proteomics* **4:** (5) 1421.

Klemm M, Schrattenholz A. 2004. Neurotoxicity of active compounds - Establishment of hESC-lines and proteomics technologies for human embryo- and neurotoxicity screening and biomarker identification. *ALTEX* **21:** (Suppl) 41.

Korn EL, Habermann JK, Upender MB, Ried T, McShane LM. 2004. Objective method of comparing DNA microarray image analysis systems. *Biotechniques* **36:** (6) 960.

Kuttner G, Giessmann E, Wessner H, Scholz C, Reinhardt D, Winkler K, Marx U, Hohne W. 2004. Linker peptide and affinity tag for detection and purification of single-chain Fv fragments. *Biotechniques* **36:** (5) 864.

Lee YH, Kim MS, Choie WS, Min HK, Lee SW. 2004. Highly informative proteome analysis by combining improved *N*-terminal sulfonation for *de novo* peptide sequencing and online capillary reverse-phase liquid chromatography/tandem mass spectrometry. *Proteomics* **4:** (6) 1684.

Copyright © 2005 John Wiley & Sons, Ltd. 

Liu Z, Pawliszyn J. 2004. Capillary isoelectric focusing with laser-induced fluorescence whole column imaging detection as a tool to monitor reactions of proteins. *J Proteome Res* **3:** (3) 567.
Lynch M, Mosher C, Huff J, Nettikadan S, Johnson J, Henderson E. 2004. Functional protein nanoarrays for biomarker profiling. *Proteomics* **4:** (6) 1695.
Marshall J, Jankowski A, Furesz S, Kireeva I, Barker L, Dombrovsky M, Zhu WM, Jacks K, Ingratta L, Bruin J, Kristensen E, Zhang R, Stanton E, Takahashi M, Jackowski G. 2004. Human serum proteins preseparated by electrophoresis or chromatography followed by tandem mass spectrometry. *J Proteome Res* **3:** (3) 364.
Maurer MH. 2004. Simple method for three-dimensional representation of 2-DE spots using a spreadsheet program. *J Proteome Res* **3:** (3) 665.
Mecham BH, Klus GT, Strovel J, Augustus M, Byrne D, Bozso P, Wetmore DZ, Mariani TJ, Kohane IS, Szallasi Z. 2004. Sequence-matched probes produce increased cross-platform consistency and more reproducible biological results in microarray-based gene expression measurements - Online article. no. e74. *Nucleic Acids Res* **32:** (9) e74.
Meng FY, Du Y, Miller LM, Patrie SM, Robinson DE, Kelleher NL. 2004. Molecular-level description of proteins from *Saccharomyces cerevisiae* using quadrupole FT hybrid mass spectrometry for top down proteomics. *Anal Chem* **76:** (10) 2852.
Metodiev MV, Timanova A, Stone DE. 2004. Differential phosphoproteome profiling by affinity capture and tandem matrix-assisted laser desorption/ionization mass spectrometry. *Proteomics* **4:** (5) 1433.
Migneault I, Dartiguenave C, Vinh J, Bertrand MJ, Waldron KC. 2004. Comparison of two glutaraldehyde immobilization techniques for solid-phase tryptic peptide mapping of human hemoglobin by capillary zone electrophoresis and mass spectrometry. *Electrophoresis* **25:** (9) 1367.
Pesek JJ, Matyska MT, Dawson GB, Chen JIC, Boysen RI, Hearn MTW. 2004. Open-tubular electrochromatographic characterization of synthetic peptides. *Electrophoresis* **25:** (9) 1211.
Ramstrom M, Bergquist J. 2004. Miniaturized proteomics and peptidomics using capillary liquid separation and high mass spectrometry. *FEBS Lett* **567:** (1) 92.
Ravichandran V, Vasquez GB, Srivastava S, Verma M, Petricoin E, Lubell J, Sriram RD, Barker PE, Gilliland GL. 2004. Data standards for proteomics: Mitochondrial two-dimensional polyacrylamide gel electrophoresis data as a model system. *Mitochondrion* **3:** (6) 327.
Rill RL, Al-Sayah MA. 2004. Peptide separations by slab gel electrophoresis in pluronic F127 polymer liquid crystals. *Electrophoresis* **25:** (9) 1249.
Sartor M, Schwanekamp J, Halbleib D, Mohamed I, Karyala S, Medvedovic M, Tomlinson CR. 2004. Microarray results improve significantly as hybridization approaches equilibrium. *Biotechniques* **36:** (5) 790.
Stewart II, Zhao L, Le Bihan T, Larsen B, Scozzaro S, Figeys D, Mao GD, Ornatsky O, Dharsee M, Orsi C, Ewing R, Goh R. 2004. The reproducible acquisition of comparative liquid chromatography/tandem mass spectrometry data from complex biological samples. *Rapid Commun Mass Spectrom* **18:** (15) 1697.
Striebel HM, Schellenberg P, Grigaravicius P, Greulich KO. 2004. Readout of protein microarrays using intrinsic time resolved UV fluorescence for label-free detection. *Proteomics* **4:** (6) 1703.
Syka JEP, Marto JA, Bai DL, Horning S, Senko MW, Schwartz JC, Ueberheide B, Garcia B, Busby S, Muratore T, Shabanowitz J, Hunt DF. 2004. Novel linear quadrupole ion trap/FT mass spectrometer: Performance characterization and use in the comparative analysis of histone H3 post-translational modifications. *J Proteome Res* **3:** (3) 621.
Szaniszlo P, Wang N, Sinha M, Reece LM, Van Hook JW, Luxon BA, Leary JF. 2004. Getting the right cells to the array: Gene expression microarray analysis of cell mixtures and sorted cells. *Cytometry* **59A:** (2) 191.
Tian GW, Mohanty A, Chary SN, Li SJ, Paap B, Drakakaki G, Kopec CD, Li JX, Ehrhardt D, Jackson D, Rhee SY, Raikhel NV, Citovsky V. 2004. High-throughput fluorescent tagging of full-length *Arabidopsis* gene products in planta. *Plant Physiol* **135:** (1) 25.
Wenner BR, Lynn BC. 2004. Factors that affect ion trap data-dependent MS/MS in proteomics. *J Am Soc Mass Spectrom* **15:** (2) 150.
Woodward AM, Rowland JJ, Kell DB. 2004. Fast automatic registration of images using the phase of a complex wavelet transform: Application to proteome gels. *Analyst* **129:** (6) 542.
Ye ML, Hu S, Schoenherr RM, Dovichi NJ. 2004. On-line protein digestion and peptide mapping by capillary electrophoresis with post- column labeling for laser-induced fluorescence detection. *Electrophoresis* **25:** (9) 1319.
Yoo YS, Regnier FE. 2004. Proteomic analysis of carbonylated proteins in two-dimensional gel electrophoresis using avidin-fluorescein affinity staining. *Electrophoresis* **25:** (9) 1334.
Zeghouf M, Li J, Butland G, Borkowska A, Canadien V, Richards D, Beattie B, Emili A, Greenblatt JF. 2004. Sequential Peptide Affinity (SPA) system for the identification of mammalian and bacterial protein complexes. *J Proteome Res* **3:** (3) 463.

### I6 Bioinformatics

Ahren D, Troein C, Johansson T, Tunlid A. 2004. PHOREST: A web-based tool for comparative analyses of expressed sequence tag data. *Mol Ecol* **4:** (2) 311.
Akmaev VR, Wang CJ. 2004. Correction of sequence-based artifacts in serial analysis of gene expression. *Bioinformatics* **20:** (8) 1254.
Arakaki AK, Zhang Y, Skolnick J. 2004. Large-scale assessment of the utility of low-resolution protein structures for biochemical function assignment. *Bioinformatics* **20:** (7) 1087.
Arthur JW, Wilkins MR. 2004. Using proteomics to mine genome sequences. *J Proteome Res* **3:** (3) 393.
Aung Z, Tan KL. 2004. Rapid 3D protein structure database searching using information retrieval techniques. *Bioinformatics* **20:** (7) 1045.
Azad RK, Borodovsky M. 2004. Effects of choice of DNA sequence model structure on gene identification accuracy. *Bioinformatics* **20:** (7) 993.
Barra V. 2004. Analysis of gene expression data using functional principal components. *Comput Methods Program Biomed* **75:** (1) 1.
Berezin C, Glaser F, Rosenberg J, Paz I, Pupko T, Fariselli P, Casadio R, Ben-Tal N. 2004. ConSeq: The identification of functionally and structurally important residues in protein sequences. *Bioinformatics* **20:** (8) 1322.
Birney E, Clamp M, Durbin R. 2004. GeneWise and Genomewise. *Genome Res* **14:** (5) 988.
Brendel V, Xing LQ, Zhu W. 2004. Gene structure prediction from consensus spliced alignment of multiple ESTs matching the same genomic locus. *Bioinformatics* **20:** (7) 1157.
Cai YD, Chou KC. 2004. Predicting subcellular localization of proteins in a hybridization space. *Bioinformatics* **20:** (7) 1151.
Cai YD, Doig AJ. 2004. Prediction of *Saccharomyces cerevisiae* protein functional class from functional domain composition. *Bioinformatics* **20:** (8) 1292.
Chesler EJ, Lu L, Wang JT, Williams RW, Manly KF. 2004. WebQTL: Rapid exploratory analysis of gene expression and genetic networks for brain and behavior. *Nat Neurosci* **7:** (5) 485.
Choi KP, Zeng FF, Zhang LX. 2004. Good spaced seeds for homology search. *Bioinformatics* **20:** (7) 1053.
Creasy DM, Cottrell JS. 2004. Unimod: Protein modifications for mass spectrometry. *Proteomics* **4:** (6) 1534.
Crooks GE, Hon G, Chandonia JM, Brenner SE. 2004. WebLogo: A sequence logo generator. *Genome Res* **14:** (6) 1188.
Cuff JA, Coates GMP, Cutts TJR, Rae M. 2004. The Ensembl computing architecture. *Genome Res* **14:** (5) 971.
Curtis RK, Brand MD. 2004. Analysing microarray data using modular regulation analysis. *Bioinformatics* **20:** (8) 1272.
Dupont A, Tokarski C, Dekeyzer O, Guilhot AL, Amouyel P, Rolando C, Pinet F. 2004. Two-dimensional maps and databases of the human macrophage proteome and secretome. *Proteomics* **4:** (6) 1761.
Ebedes J, Datta A. 2004. Multiple sequence alignment in parallel on a workstation cluster. *Bioinformatics* **20:** (7) 1193.
Edgar RC, Sjolander K. 2004. COACH: Profile-profile alignment of protein families using hidden Markov models. *Bioinformatics* **20:** (8) 1309.
Eyras E, Caccamo M, Curwen V, Clamp M. 2004. ESTGenes: Alternative splicing from ESTs in Ensembl. *Genome Res* **14:** (5) 976.
Faller D, Reinheckel T, Wenzler D, Hagemann S, Xiao K, Honerkamp J, Peters C, Dandekar T, Timmer J. 2004. An open source protein gel

Copyright © 2005 John Wiley & Sons, Ltd. 

documentation system for proteome analyses. *J Chem Inform Comput Sci* **44:** (1) 168.
Farriol-Mathis N, Garavelli JS, Boeckmann B, Duvaud S, Gasteiger E, Gateau A, Veuthey AL, Bairoch A. 2004. Annotation of post-translational modifications in the Swiss-Prot knowledge base. *Proteomics* **4:** (6) 1537.
Flibotte S, Chiu R, Fjell C, Krzywinski M, Schein JE, Shin H, Marra MA. 2004. Automated ordering of fingerprinted clones. *Bioinformatics* **20:** (8) 1264.
Garavelli JS. 2004. The RESID Database of Protein Modifications as a resource and annotation tool. *Proteomics* **4:** (6) 1527.
Halling-Brown M, Sansom C, Moss DS, Elgar G, Edwards YJK. 2004. A Fugu-Human Genome Synteny Viewer: Web software for graphical display and annotation reports of synteny between fugu genomic sequence and human genes. *Nucleic Acids Res* **32:** (8) 2618.
Hashimoto RF, Kim S, Shmulevich I, Zhang W, Bittner ML, Dougherty ER. 2004. Growing genetic regulatory networks from seed genes. *Bioinformatics* **20:** (8) 1241.
Hayashi D, Imai Y, Morita H, Fujita H, Monzen K, Harada T, Nojiri T, Yamazaki T, Yamazaki T, Nagai R. 2004. Development of a pioneering clinical support system utilizing information technology - Clinical informatics and genome analysis. *Jpn Heart J* **45:** (2) 315.
Heintz N. 2004. Gene expression nervous system atlas (GENSAT). *Nat Neurosci* **7:** (5) 483.
Hornbeck PV, Chabra I, Kornhauser JM, Skrzypek E, Zhang B. 2004. PhosphoSite: A bioinformatics resource dedicated to physiological protein phosphorylation. *Proteomics* **4:** (6) 1551.
Iossifov I, Krauthammer M, Friedman C, Hatzivassiloglou V, Bader JS, White KP, Rzhetsky A. 2004. Probabilistic inference of molecular networks from noisy data sources. *Bioinformatics* **20:** (8) 1205.
Izaguirre JA, Chaturvedi R, Huang C, Cickovski T, Coffland J, Thomas G, Forgacs G, Alber M, Hentschel G, Newman SA *et al*. 2004. CompuCell, a multi-model framework for simulation of morphogenesis. *Bioinformatics* **20:** (7) 1129.
James AC, Veitch JG, Zareh AR, Triche T. 2004. Sensitivity and specificity of five abundance estimators for high-density oligonucleotide microarrays. *Bioinformatics* **20:** (7) 1060.
Kim HK, Jung YJ, Choi WC, Ryu HS, Oh TK, Lee JK. 2004. Sequence-based approach to finding functional lipases from microbial genome databases. *FEMS Microbiol Lett* **235:** (2) 349.
Kim J, Chung HJ, Park CH, Park WY, Kim JH. 2004. ChromoViz: Multimodal visualization of gene expression data onto chromosomes using scalable vector graphics. *Bioinformatics* **20:** (7) 1191.
Kim WK, Bolser DM, Park JH. 2004. Large-scale co-evolution analysis of protein structural interlogues using the global protein structural interactome map (PSIMAP). *Bioinformatics* **20:** (7) 1138.
King RD, Wise H, Clare A. 2004. Confirmation of data mining based predictions of protein function. *Bioinformatics* **20:** (7) 1110.
Krasnogor N, Pelta DA. 2004. Measuring the similarity of protein structures by means of the universal similarity metric. *Bioinformatics* **20:** (7) 1015.
Krebs WG, Bourne PE. 2004. Statistically rigorous automated protein annotation. *Bioinformatics* **20:** (7) 1066.
Kumar Y, Khachane A, Belwal M, Das SJ, Somsundaram K, Tatu U. 2004. ProteoMod: A new tool to quantitate protein post-translational modifications. *Proteomics* **4:** (6) 1672.
Lepre J, Rice JJ, Tu YH, Stolovitzky G. 2004. Genes@Work: An efficient algorithm for pattern discovery and multivariate feature selection in gene expression data. *Bioinformatics* **20:** (7) 1033.
Li X, Rao SQ, Wang YD, Gong BS. 2004. Gene mining: A novel and powerful ensemble decision approach to hunting for disease genes using microarray expression profiling. *Nucleic Acids Res* **32:** (9) 2685.
Lin M, Wei LJ, Sellers WR, Lieberfarb M, Wong WH, Li C. 2004. dChipSNP: Significance curve and clustering of SNP-array-based loss-of-heterozygosity data. *Bioinformatics* **20:** (8) 1233.
Lomax J, McCray AT. 2004. Mapping the Gene Ontology into the Unified Medical Language System. *Comp Funct Genom* **5:** (4) 354.
Mandava S, Makowski L, Devarapalli S, Uzubell J, Rodi DJ. 2004. RELIC - A bioinformatics server for combinatorial peptide analysis and identification of protein-ligand interaction sites. *Proteomics* **4:** (5) 1439.
Medvedovic M, Yeung KY, Bumgarner RE. 2004. Bayesian mixture model based clustering of replicated microarray data. *Bioinformatics* **20:** (8) 1222.
Meyers BC, Lee DK, Vu TH, Tej SS, Edberg SB, Matvienko M, Tindell LD. 2004. *Arabidopsis* MPSS. An online resource for quantitative expression analysis. *Plant Physiol* **135:** (2) 801.
Mignone F, Horner DS, Pesole G. 2004. WebVar: A resource for the rapid estimation of relative site variability from multiple sequence alignments. *Bioinformatics* **20:** (8) 1331.
Minakuchi Y, Ito M, Kohara Y. 2004. SPI: A tool for incorporating gene expression data into a four-dimensional database of *Caenorhabditis elegans* embryogenesis. *Bioinformatics* **20:** (7) 1097.
Montgomery S, Astakhova T, Bilenky M, Birney E, Fu T, Hassel M, Melsopp C, Rak M, Robertson A, Sleumer M *et al*. 2004. Sockeye: A 3D environment for comparative genomics. *Genome Res* **14:** (5) 956.
Olsen L, Hansen M, Ekstrom CT, Troelsen JT, Olsen J. 2004. CVD: The intestinal crypt/villus *in situ* hybridization database. *Bioinformatics* **20:** (8) 1327.
Ovcharenko I, Boffelli D, Loots GG. 2004. eShadow: A tool for comparing closely related sequences. *Genome Res* **14:** (6) 1191.
Pleissner KP, Eifert T, Buettner S, Schmidt F, Boehme M, Meyer TF, Kaufmann SHE, Jungblut PR. 2004. Web-accessible proteome databases for microbial research. *Proteomics* **4:** (5) 1305.
Potter SC, Clarke L, Curwen V, Keenan S, Mongin E, Searle SMJ, Stabenau A, Storey R, Clamp M. 2004. The Ensembl analysis pipeline. *Genome Res* **14:** (5) 934.
Searle SMJ, Gilbert J, Iyer V, Clamp M. 2004. The Otter annotation system. *Genome Res* **14:** (5) 963.
Shah NH, Fedoroff NV. 2004. CLENCH: A program for calculating CLuster ENriCHment using the Gene Ontology. *Bioinformatics* **20:** (7) 1196.
Sharma R, Maheshwari JK, Dash D, Brahmachari SK. 2004. Recognition and analysis of protein-coding genes in severe acute respiratory syndrome associated coronavirus. *Bioinformatics* **20:** (7) 1074.
Shmuely H, Dinitz E, Dahan I, Eichler J, Fischer D, Shaanan B. 2004. Poorly conserved ORFs in the genome of the archaea *Halobacterium* sp NRC-1 correspond to expressed proteins. *Bioinformatics* **20:** (8) 1248.
Small I, Peeters N, Legeai F, Lurin C. 2004. Predotar: A tool for rapidly screening proteomes for *N*-terminal targeting sequences. *Proteomics* **4:** (6) 1581.
Stabenau A, McVicker G, Melsopp C, Proctor G, Clamp M, Birney E. 2004. The Ensembl core software libraries. *Genome Res* **14:** (5) 929.
Stalker J, Gibbins B, Meidl P, Smith J, Spooner W, Hotz HR, Cox AV. 2004. The Ensembl Web site: Mechanics of a genome browser. *Genome Res* **14:** (5) 951.
Terai G, Takagi T. 2004. Predicting rules on organization of *cis*-regulatory elements, taking the order of elements into account. *Bioinformatics* **20:** (7) 1119.
Traxler E, Bayer E, Stockl J, Mohr T, Lenz C, Gerner C. 2004. Towards a standardized human proteome database: Quantitative proteome profiling of living cells. *Proteomics* **4:** (5) 1314.
Weckx S, De Rijk P, Van Broeckhoven C, Del-Favero J. 2004. SSHSuite: An integrated software package for analysis of large-scale suppression subtractive hybridization data. *Biotechniques* **36:** (6) 1043.
Wurgler-Murphy SM, King DM, Kennelly PJ. 2004. The phosphorylation site database: A guide to the serine-, threonine-, and/or tyrosine-phosphorylated proteins in prokaryotic organisms. *Proteomics* **4:** (6) 1562.
Yip YL, Scheib H, Diemand AV, Gattiker A, Famiglietti LM, Gasteiger E, Bairoch A. 2004. The Swiss-Prot variant page and the ModSNP database: A resource for sequence and structure information on human protein variants. *Hum Mutat* **23:** (5) 464.
Yu H, Yoo AS, Greenwald I. 2004. Cluster Analyzer for Transcription Sites (CATS): A C++-based program for identifying clustered transcription factor binding sites. *Bioinformatics* **20:** (7) 1198.
Yu HY, Luscombe NM, Lu HX, Zhu XW, Xia Y, Han JDJ, Bertin N, Chung S, Vidal M, Gerstein M. 2004. Annotation transfer between genomes: Protein-protein interologs and protein-DNA regulogs. *Genome Res* **14:** (6) 1107.
Zhang LD, Yuan DJ, Yu SW, Li ZG, Cao YF, Miao ZQ, Qian HM, Tang KX. 2004. Preference of simple sequence repeats in coding and non-coding regions of *Arabidopsis thaliana*. *Bioinformatics* **20:** (7) 1081.

Copyright © 2005 John Wiley & Sons, Ltd. 
